# Dopamine neuron-derived IGF-1 controls dopamine neuron firing, skill learning, and exploration

**DOI:** 10.1073/pnas.1806820116

**Published:** 2019-02-11

**Authors:** Alessandro Pristerà, Craig Blomeley, Emanuel Lopes, Sarah Threlfell, Elisa Merlini, Denis Burdakov, Stephanie Cragg, François Guillemot, Siew-Lan Ang

**Affiliations:** ^a^The Francis Crick Institute, London NW1 1AT, United Kingdom;; ^b^Department of Physiology, Anatomy and Genetics, University of Oxford, Oxford OX1 3QX, United Kingdom;; ^c^Oxford Parkinson’s Disease Centre, University of Oxford, Oxford OX1 3PT, United Kingdom

**Keywords:** IGF-1, firing, behavior, dopamine, somatodendritic

## Abstract

Midbrain dopamine neurons, which can be regulated by neuropeptides and hormones, play a fundamental role in controlling cognitive processes, reward mechanisms, and motor functions. The hormonal actions of insulin-like growth factor 1 (IGF-1) produced by the liver have been well described, but the role of neuronally derived IGF-1 remains largely unexplored. We discovered that dopamine neurons secrete IGF-1 from the cell bodies following depolarization, and that IGF-1 controls release of dopamine in the ventral midbrain. In addition, conditional deletion of dopamine neuron-derived IGF-1 in adult mice leads to decrease of dopamine content in the striatum and deficits in dopamine neuron firing and causes reduced spontaneous locomotion and impairments in explorative and learning behaviors. These data identify that dopamine neuron-derived IGF-1 acts as a regulator of dopamine neurons and regulates dopamine-mediated behaviors.

## Significance

Midbrain dopamine neurons play a role in motivational and cognitive control of behavior. In addition, they regulate motor functions. Dysregulation of dopamine neurons has been linked to depression, schizophrenia, and addiction and their degeneration is causal to Parkinson’s disease. Peripheral hormones have been shown to regulate dopamine neurons functions. Insulin-like growth factor 1 (IGF-1) is a hormone mainly produced in the liver. With this study we discovered that midbrain dopamine neurons synthesize and release IGF-1 in an activity dependent manner. In addition, dopamine neuron-derived IGF-1 modulates dopamine synthesis and dopamine neuron firing and ultimately it controls dopamine-dependent behaviors. This study highlights the neuromodulatory role of neuron-derived IGF-1 and its role in shaping dopamine transmission in the brain.

Midbrain dopamine (mDA) neurons play a fundamental role in a multitude of cognitive processes, including reward processing, learning, decision making, and motivation to engage in goal-orientated behaviors (like eating and drinking) (1, 2). mDA neurons are organized into nuclei in the ventral midbrain, with the two major ones being the ventral tegmental area (VTA) and substantia nigra pars compacta (SNc). VTA and SNc neurons send long projection axons to the ventral striatum, prefrontal cortex, and dorsal striatum. Dysregulation of DA transmission in humans is associated with cognitive impairments such as schizophrenia, anxiety, and depression (3). In addition, mDA neurons control motor function; their degeneration in the SNc underlies movement problems in Parkinson’s disease (4). A detailed knowledge of the biology and functioning of mDA neurons is therefore important to understand both physiological and pathological states.

Insulin-like growth factor 1 (IGF-1) is a pleiotropic 70 amino-acid residues long hormone that is mainly secreted from the liver, under the influence of the growth hormone (5). Its biological actions are dependent on the developmental stage, its concentration, time course of action, and target cell type. IGF-1 has been shown to promote differentiation and maturation of neurons, and IGF-1 whole-body KO and IGF-1 receptor (IGF-1R) brain-specific conditional KO mice have reduced brain size (6, 7). In the adult mouse brain, IGF-1 modulates neuronal plasticity and positively promotes neurogenesis (8). In humans, IGF-1 deficiency causes dwarfism (5) and mutations in the *Igf1* gene cause growth failure in utero, microcephaly, and mental retardation postnatally (9).

In addition to its production by the liver, IGF-1 can also be synthesized in the CNS by neurons. While the role of peripheral IGF-1 secreted mainly from the liver has been extensively studied, the role of neuronally derived IGF-1 is only beginning to be uncovered. Neuronal IGF-1 has been shown to affect neuronal function by modulating excitability and synaptic connections. For example, Cao et al. (10) demonstrated that IGF-1 is secreted from dendrites and cell bodies of mitral neurons in the olfactory bulb following depolarization. Further investigation showed that IGF-1 modulates synaptic plasticity of mitral cells during social learning in an autocrine fashion (11). IGF-1 has also been recently reported to be highly up-regulated in vasoactive intestinal peptide (VIP)-expressing interneurons of the cortex following sensory experience. VIP neuron-derived IGF-1 acutely promotes inhibition onto VIP neurons in a cell-autonomous manner (12). Interestingly, *Igf1* transcripts have been found in mDA neurons, preferentially in the SNc, both by microarray (in adult) (13) and single-cell RT-qPCR (at postnatal day 4) (14), yet whether and which mDA neurons express IGF-1 protein was not explored.

Studies have shown that the activity of mDA neurons is regulated by neuropeptides secreted from afferent neurons (15) and hormones from the periphery (16, 17). The sensitivity of mDA neurons to IGF-1 signaling has been supported by the demonstration that ectopic application of IGF-1 promotes survival of mDA neurons following a toxic insult in vitro (13) and in vivo (18). Despite the notion that mDA neurons themselves may be a source of IGF-1 in the adult brain and that neuronally derived IGF-1 can act as a neuromodulator, no studies to date have explored the role of mDA neuron-derived IGF-1. Considering the involvement of DA signaling in shaping cognitive and motor function, both in physiological and pathological scenarios, we believe that a detailed understanding of DA neuron modulation is of great importance. In this study, we show that mDA neurons synthesize and secrete IGF-1 from the cell body following depolarization. We also demonstrate that IGF-1 controls striatal DA levels, local DA release in the midbrain, and DA neuron firing. Moreover, elimination of DA neuron-derived IGF-1 in mice is sufficient to cause hypoactivity, reduced exploratory behavior, and impaired motor learning skills.

## Results

### IGF-1 and IGF-1R Are Expressed in the SNc and VTA.

**Fig. 1.**
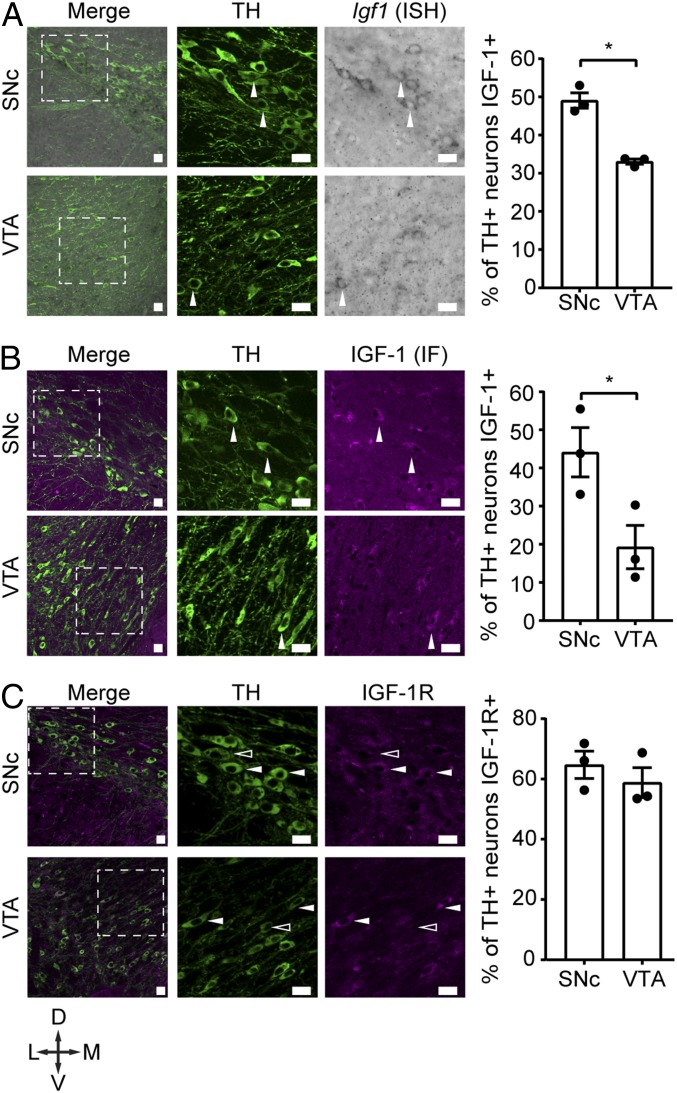
IGF-1 and IGF-1R are expressed in the SNc and VTA. (*A*) *Igf1* transcripts, detected by ISH, are detected in TH-positive neurons in the SNc and VTA. Merge shows the overlap of immunofluorescence for TH and bright-field imaging for *Igf1* ISH. White arrowheads point at TH-positive neurons positive for *Igf1* ISH probe. Percentage of TH-positive neurons expressing *Igf1* transcripts is higher in the SNc than in the VTA (*n* = 3 mice, *P* = 0.0017, *t* = 7.502 df = 4; two-tailed unpaired Student’s *t* test). (*B*) IGF-1 protein immunoreactivity, detected by immunofluorescence, is detectable in TH-positive neurons in the SNc and VTA. Merge shows the overlap of immunofluorescence for TH and IGF-1. White arrowheads point at neurons double-positive for TH and IGF-1. Percentage of TH-positive neurons expressing IGF-1 protein is higher in the SNc than in the VTA (*n* = 3 mice, *P* = 0.0446, *t* = 2.888 df = 4; two-tailed unpaired Student’s *t* test). (*C*) IGF-1R protein immunoreactivity, detected by immunofluorescence, is present in TH-positive neurons in the SNc and VTA. Merge shows the overlap of immunofluorescence for TH and IGF-1R. White arrowheads point at neurons double-positive for TH and IGF-1R, open arrowheads point at TH positive neurons, negative for IGF-1R immunoreactivity. Percentage of TH-positive neurons with IGF-1R protein is the same between SNc and VTA (*n* = 3 mice, *P* = 0.4282, *t* = 0.8807 df = 4; two-tailed unpaired Student’s *t* test). Dotted squares in the merge pictures are magnified and split for clarity. Crossed arrows show image orientation. D, dorsal; L, lateral; M, medial; V, ventral. Graphs show mean ± SEM, together with individual values. *P < 0.05. (Scale bars: 20 μm.)

In this study, we set out to investigate the expression and role of mDA neuron-derived IGF-1 on mDA neuron activity and their dependent behaviors. Using in situ hybridization (ISH) and immunofluorescence on brain sections, we found that *Igf1* transcripts and protein are distributed in a heterogeneous manner in the VTA and SNc throughout the rostral–caudal extent of the ventral midbrain (Fig. 1 *A* and *B* and *SI Appendix*, Fig. S1*A*). Cell counting revealed a larger fraction of mDA neurons expressing both DA neuron marker tyrosine hydroxylase (TH) and IGF-1 in the SNc than in the VTA. (Fig. 1 *A* and *B*, white arrowheads and graphs). IGF-1 immunoreactivity was detected at the level of the cell bodies but was not found to be associated with TH-positive dendrites in the substantia nigra pars reticulata or TH-positive axons in the striatum (*SI Appendix*, Fig. S1*B*). A negative control experiment, where the primary anti-IGF-1 antibody was omitted, showed nonspecific background staining, whereas IGF-1 immunoreactivity was only detected in the presence of the primary antibody (*SI Appendix*, Fig. S1*C*).

IGF-1 exerts its biological actions mostly by binding and activating its high-affinity receptor, IGF-1R (8). To determine the proportion of mDA neurons capable of responding to IGF-1, we quantified the percentage of TH-positive mDA neurons with immunoreactivity for the IGF-1R. We found that IGF-1R immunoreactivity was detectable in about 60% of TH-positive neurons in the SNc and VTA (Fig. 1*C*). These findings suggest the possibility of autocrine/paracrine signaling of IGF-1 in mDA neurons, and that SNc DA neurons may be exposed to higher levels of locally synthesized IGF-1 than VTA neurons.

### IGF-1 Is Released in an Activity-Dependent Manner.

Since neuropeptides usually require neuronal activity to be released (19, 20) we examined whether IGF-1 is released by mDA neurons in an activity-dependent manner. To test this hypothesis, we modulated the activity of mouse primary mDA neurons in vitro using pharmacological treatments and quantified the intracellular immunolabeling of IGF-1, as a proxy of IGF-1 release.

**Fig. 2.**
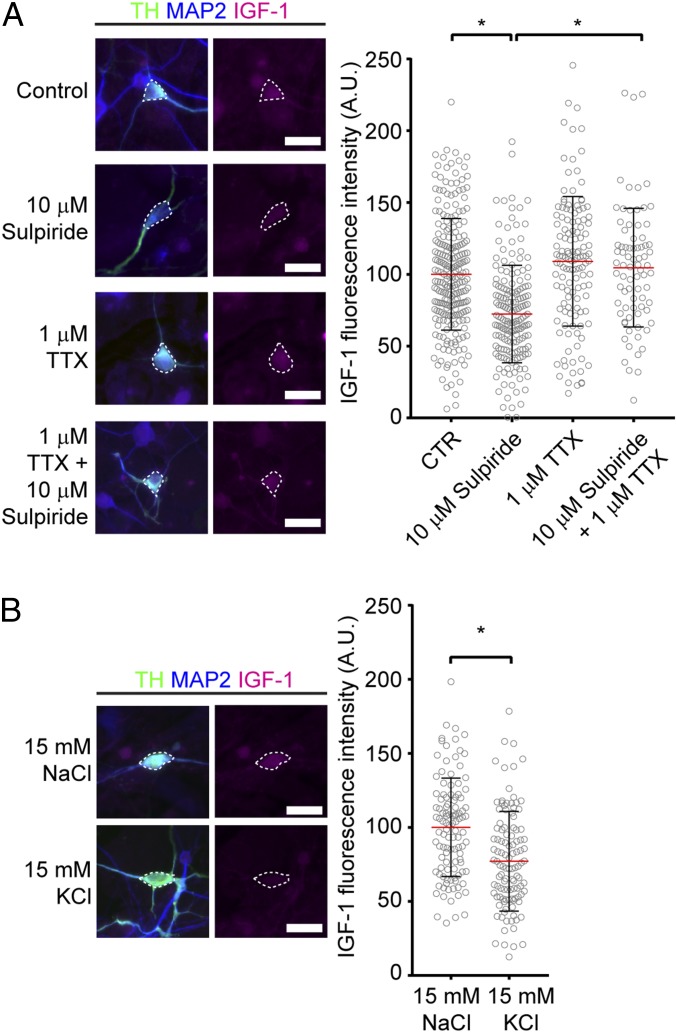
IGF-1 is released in an activity-dependent manner. Images in *A* and *B* show representative immunofluorescence of mDA neurons (TH−, MAP2-positive) expressing IGF-1, following different treatments. IGF-1 intracellular fluorescence intensity in the cell bodies (dotted ROIs in the images) was quantified. (*A*) Graph shows mean ± SD, together with distribution of individual neuron following 10 μM sulpiride, 1 μM TTX, or 10 μM sulpiride + 1 μM TTX [CTR and 10 μM sulpiride *n* = 6 cultures, 1 μM TTX and 10 μM sulpiride + 1 μM TTX *n* = 3 cultures. *P* CTR vs. 10 μM sulpiride = <0.0001, *P* 10 μM sulpiride vs. 10 μM sulpiride + 1 μM TTX = <0.0001, *P* CTR vs. 1 μM TTX = 0.1520; *F*_(3,__624)_ = 28.04; one-way ANOVA with Tukey’s correction for multiple comparisons]. (*B*) Graph shows mean ± SD, together with distribution of individual neuron following 15 mM NaCl or 15 mM KCl treatments (*n* = 3 cultures, *P* ≤ 0.0001, *t* = 5.086 df = 219; two-tailed unpaired Student’s *t* test). *P < 0.05. (Scale bars: 20 μm.)

We generated primary cultures from E16 ventral midbrains that contained mDA neurons coexpressing TH and D2 receptor (D2R) (*SI Appendix*, Fig. S2*A*). As expected from our findings in brain sections, IGF-1 protein was abundantly expressed in the cell bodies of cultured mDA neurons [Fig. 2, dotted regions of interest (ROIs)]. TH, IGF-1, and microtubule associated protein 2 (MAP2) triple labeling was used to distinguish IGF-1 localization in dendritic vs. axonal processes in mDA neurons, since MAP2 labels the dendritic and not the axonal processes (21). Similar to the in vivo analysis, IGF-1 immunolabeling was not detected in the axons (TH+ MAP2−); however, it was also observed in some dendrites (TH+ MAP2+) of mDA neurons (*SI Appendix*, Fig. S2*B*).

To assess if IGF-1 is released from the soma in an activity-dependent manner, we quantified the intensity of IGF-1 immunolabeling in cultured TH-expressing neurons following different pharmacological treatments. As inactivation of D2 receptors increases the firing activity of mDA neurons in situ (22, 23), we used sulpiride, an antagonist of D2R (24), to increase DA neuron excitability. Indeed, 1-h treatment with 10 μM sulpiride apparently increased DA neuron excitability, as measured using the immediate early protein Fos (*SI Appendix*, Fig. S2*C*); 10 μM sulpiride treatment for 1 h led to a reduction of intracellular IGF-1 immunolabeling, consistent with an extracellular release of IGF-1 (Fig. 2*A*, ROIs defined by dotted line and graph). In contrast, TH fluorescence levels did not change in these neurons, compared with controls (CTRs). These results suggest that the decrease in IGF-1 immunolabeling is not due to nonspecific diffusion of fluorescence signals or an experimental artifact (*SI Appendix*, Fig. S2*D*). Tetrodotoxin (TTX) treatment, which blocks voltage-gated sodium channels and action potential generation, did not have a significant effect on IGF-1 immunolabeling when used alone but abolished sulpiride action (Fig. 2*A*). Taken together these data suggest that the reduction in intracellular IGF-1 levels mediated by sulpiride is due to effects on electrical activity. We confirmed the putative activity-dependent release of IGF-1 by depolarizing DA neuron cultures for 1 h with 15 mM KCl, compared with 15 mM NaCl as a control for osmolarity. KCl treatment led to a decrease in IGF-1 intracellular immunolabeling (Fig. 2*B*, ROIs defined by dotted line and graph) consistent with an activity-dependent release of IGF-1. We further investigated the mechanism of IGF-1 intracellular decrease to test whether this was due to IGF-1 release. Since mDA neurons spontaneously fire in vitro (25), we first determined whether a potential reduction in neuronal excitability in cultured cells by the D2 agonist quinpirole regulated IGF-1 release. Similar to TTX treatment, 1 μM quinpirole treatment for 1 h did not affect IGF-1 intracellular immunolabeling levels, suggesting that basal IGF-1 release is negligible (*SI Appendix*, Fig. S3*A*). To confirm that sulpiride-mediated decrease in intracellular IGF-1 is due to exocytosis, we treated mDA neurons with sulpiride, with or without the exocytotic blocker EXO1 (26); 100 μM EXO1 abolished the effect of sulpiride on IGF-1 intracellular level, suggesting that the sulpiride-mediated reduction in IGF-1 intracellular level is due to exocytosis of IGF-1 (*SI Appendix*, Fig. S3*B*). Overall, the above data suggest that mDA neurons release IGF-1 in an activity-dependent manner and that the release of IGF-1 in unstimulated conditions is negligible in vitro.

### Exogenously Applied IGF-1 Slows Down DA Release at the Level of the Cell Body.

IGF-1 has been found to modulate synaptic transmission in the hippocampus (27, 28). We have shown that mDA neurons release IGF-1 and express IGF-1R, suggesting that IGF-1 signaling may act in an autocrine or paracrine manner in mDA neurons to control DA release. In addition to classical vesicular release from axons, mDA neurons also release DA from their cell bodies and dendrites (29, 30). Given that the distribution of IGF-1 protein is localized to the cell body and not in the axons of mDA neurons, we sought to investigate whether IGF-1 regulates release of DA from the cell body.

**Fig. 3.**
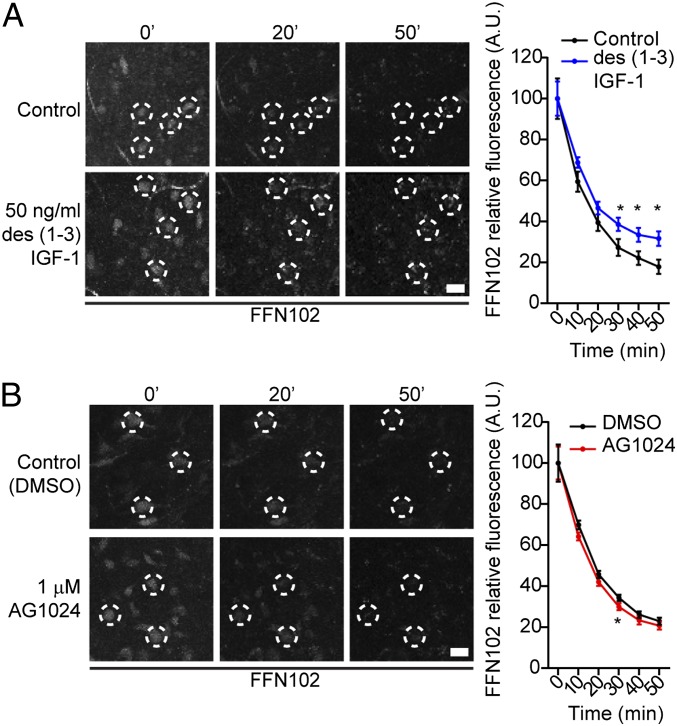
IGF-1 exposure to the ventral midbrain reduces DA release from the cell bodies of mDA neurons. FFN102 release from the cell bodies of mDA neurons was quantified over time, upon pharmacological stimulation. Panels on the left show representative FFN102 fluorescence at different time points. Dotted ROIs show examples of mDA neurons, tracked over time. Graphs show normalized FFN102 fluorescence intensity over time. (*A*) Fifty nanograms per milliliter of des(1-3)IGF-1 (IGF-1 receptor agonist), applied at T20 min, slows down DA release rate from the cell bodies [*n* = 3, P CTR vs. des(1-3)IGF-1 (T30 min, T40 min, T50 min) = <0.0001; *F*_(2,__356)_ = 3.006; two-way ANOVA with Sidak’s correction for multiple comparisons]. (*B*) One micromolar AG1024 (IGF-1 receptor antagonist), applied at T10 min, minimally increases DA release rate from the cell bodies [*n* = 3, P CTR vs. AG1024 (T30 min) = 0.0028; *F*_(3,__858)_ = 2.117; two-way ANOVA with Sidak’s correction for multiple comparisons]. Time points show mean ± 95% CI. (Scale bars: 20 μm.)

We used acute brain slices containing the ventral midbrain loaded with the fluorescent false neurotransmitter FFN102, which is used to measure DA dynamics and exhibits high selectivity for DA neuron cell bodies in the midbrain (31). To determine whether activation or inhibition of IGF-1R signaling modulates DA release, we examined the effects of the IGF-1R agonist des(1-3)IGF-1 (27) (50 ng/mL) or the IGF-1R antagonist AG1024 (28) (1 μM) on the fluorescence intensity of FFN102 over time. We found that in control conditions [i.e., slices superfused in artificial cerebrospinal fluid (ACSF) alone or ACSF/DMSO], FFN102 was spontaneously released from SNc DA neurons, in agreement with previous reports (31) (Fig. 3). In addition, we quantified the FFN102 destaining rate in the presence of 1 μM TTX, to block action potentials, and found that a small fraction of FFN102 release was TTX-dependent (15.59 ± 28.87% at min 50; mean ± SD; *n* = 3) (*SI Appendix*, Fig. S4). These data suggest that spontaneous DA release from the soma is largely independent of action potentials, in agreement with a recent report (32). Treatment with des(1-3)IGF-1 resulted in a slower rate of release of FFN102, indicating that engagement of the IGF-1R pathway by the IGF-1 agonist slows down the release of DA from the cell body (Fig. 3*A*). However, AG1024 resulted in a small increase of FFN102 rate of release at one time point, indicating that interfering with IGF-1R signaling can accelerate DA release compared with control conditions (Fig. 3*B*). These data show that activation of the IGF-1 pathway in the ventral midbrain inhibits DA release from the cell bodies of mDA neurons.

### *Igf1* Conditional KO Mice Show Reduced Activation of the IGF-1R Pathway and Are Hypodopaminergic.

**Fig. 4.**
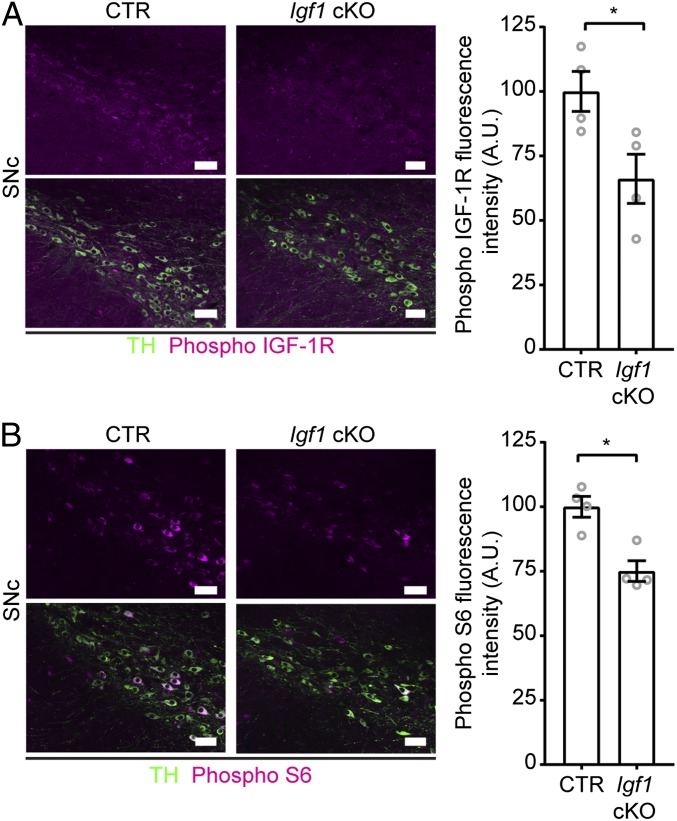
*Igf1* cKO mice are characterized by reduced IGF-1 signaling in DA neurons in the SNc. Upstream (IGF-1R) and downstream (S6 ribosomal protein) effectors of IGF-1 signaling were analyzed. (*A*) Images show representative immunofluorescence for TH and phospho IGF-1R in the SNc of CTR and *Igf1* cKO mice. Top shows phospho IGF-1R only for clarity. Phospho IGF-1R fluorescence intensity was reduced in TH-positive SNc neurons in *Igf1* cKO mice, compared with CTRs (*n* = 4, *P* = 0.0331, *t* = 2.755 df = 6; two-tailed unpaired Student’s *t* test). (*B*) Images show representative immunofluorescence for TH and phospho S6 in the SNc of CTR and *Igf1* cKO mice. Top shows phospho S6 only for clarity. Phospho S6 fluorescence intensity was reduced in TH-positive SNc neuron in *Igf1* cKO mice, compared with CTRs (*n* = 4, *P* = 0.0047, *t* = 4.379 df = 6; two-tailed unpaired Student’s *t* test). Bar graphs show mean ± SEM. *P < 0.05.

So far, we have established that IGF-1 modulates DA release from the cell bodies in brain slices. To determine whether DA neuron-derived IGF-1 has a role in vivo, we employed a genetic approach. We analyzed an inducible conditional KO mouse model, *Slc6a3*^*CreERT2/+*^*; Igf1* ^*flox/flox*^ (referred to henceforth as *Igf1* cKO). In these mice IGF-1 is deleted specifically in dopamine transporter (DAT)-expressing neurons, by Cre recombinase, upon administration of tamoxifen in adulthood. CTR mice were harboring only the floxed allele (*Igf1*^*flox/flox*^). We found, by immunofluorescence, that IGF-1 and DAT showed colocalization in the midbrain. On the contrary, they did not colocalize in the olfactory bulb, where IGF-1 is also expressed (10), or in the arcuate nucleus of the hypothalamus (*SI Appendix*, Fig. S5). A11 DA neuron group does not express DAT (33). Therefore, we used *Igf1* cKO mice to explore the role of mDA neuron-derived IGF-1. Adult *Igf1* cKO and CTR mice were analyzed from 2 to 4 weeks after tamoxifen-induced IGF-1 deletion. Colocalization analysis showed that IGF-1 protein was decreased by ∼80% at 2 weeks after tamoxifen injections (*SI Appendix*, Fig. S6*A*). We also verified that (*i*) CTR mice, homozygous for the *Igf1* floxed allele, present similar levels of *Igf1* transcripts compared with wild-type mice and are therefore not hypomorphic (*SI Appendix*, Fig. S6*B*) and (*ii*) the presence of transgene *Slc6a3*^*CreERT2*^ does not affect endogenous *Slc6a3* levels (*SI Appendix*, Fig. S6*C*). We reasoned that if IGF-1 acts in an autocrine or paracrine manner in mDA neurons, lack of IGF-1 release from these neurons in vivo may affect the IGF-1/IGF-1R signaling pathway in the ventral midbrain. We therefore quantified the immunolabeling of upstream and downstream effectors of IGF-1 signaling in mDA neurons from *Igf1* cKO and CTR mice, 2 weeks after *Igf1* conditional deletion. IGF-1R, when bound to IGF-1, autophosphorylates and phosphorylates several intracellular proteins (34). We quantified phospho IGF-1R immunolabeling and found that SNc TH-positive neurons showed reduced levels of activated IGF-1R, indicating decreased IGF-1 signaling in *Igf1* cKO compared with CTR mice (Fig. 4*A*). We also investigated downstream effectors of IGF-1 and focused on the S6 ribosomal protein, a component of the IGF-1/mammalian target of rapamycin (mTOR)/ribosomal protein S6 kinase beta-1 (p70S6K) signaling pathway (35–37). Deletion of IGF-1 resulted in a reduction of phosphorylated S6 ribosomal protein in SNc DA neurons of *Igf1* cKO mice (Fig. 4*B*). In agreement with our earlier finding that IGF-1 is localized to cell bodies and is not transported to striatal axons, we detected no change in phosphorylation level of IGF-1R in the striatum (*SI Appendix*, Fig. S7*A*). We also noted that mDA neurons in the VTA of *Igf1* cKO mice showed normal IGF-1R signaling (*SI Appendix*, Fig. S7 *B* and *C*). Together, these results establish that elimination of IGF-1 in mDA neurons reduces the engagement of the IGF-1R pathway in SNc DA neurons, indicating that IGF-1 acts in an autocrine and/or paracrine manner in these cells in vivo.

**Fig. 5.**
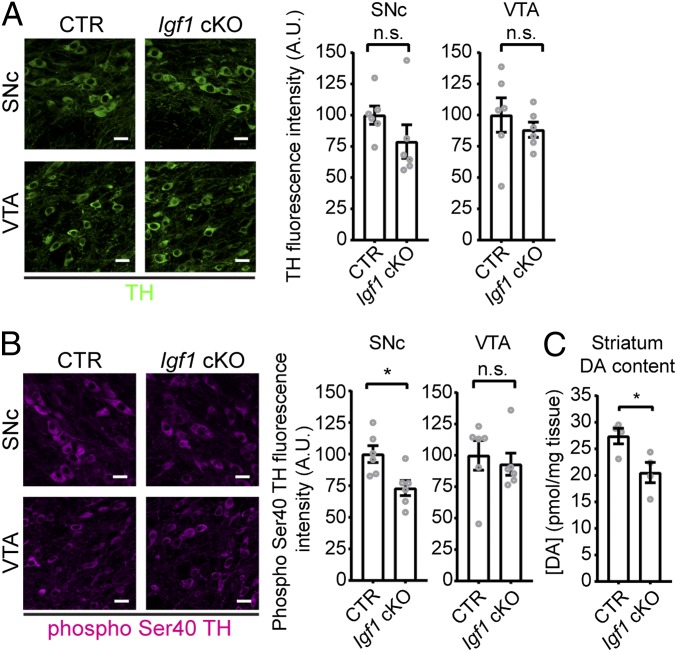
*Igf1* cKO mice show reduced levels of phospho Ser40 TH and are hypodopaminergic. (*A*) TH levels are not affected in *Igf1* cKO mice. Pictures show representative image of TH immunofluorescence in SNc and VTA of CTR and *Igf1* cKO mice. Graphs show TH fluorescence density (*n* = 6; SNc: *P* = 0.1984, *t* = 1.377 df = 10; VTA: *P* = 0.4535, *t* = 0.78 df = 10; two-tailed unpaired Student’s *t* test). (*B*) *Igf1* cKO mice show reduced levels of phospho Ser40 TH in the SNc, but not in the VTA. Pictures show representative image of phospho Ser40 TH immunofluorescence in SNc and VTA of CTR and *Igf1* cKO mice. Graphs show phospho Ser40 TH fluorescence density (*n* = 6; SNc: *P* = 0.0130, *t* = 3.014 df = 10; VTA: *P* = 0.6447, *t* = 0.4755 df = 10; two-tailed unpaired Student’s *t* test). (Scale bars: 20 μm.) (*C*) Total DA content from CTR and *Igf1* cKO striata was measured and normalized vs. mg of tissue. *Igf1* cKO mice are hypodopaminergic (*n* = 4; *P* = 0.0291, *t* = 2.852, df = 6; two-tailed unpaired Student’s *t* test). Graph bars show mean ± SEM, together with individual value of replicates.

We then examined whether reduction of IGF-1R signaling resulted in cellular defects in mDA neurons of *Igf1* cKO mice. IGF-1 has previously been shown to be neuroprotective (18), to regulate cell size (38), and to promote the enzymatic activity of TH in chromaffin cells (39). Therefore, 2 weeks after *Igf1* conditional deletion we counted mDA neurons number and analyzed their gross morphology. In addition we assessed TH protein levels and DA content. We did not observe any change in DA neuron number and soma size in either the SNc or VTA of *Igf1* cKO mice (*SI Appendix*, Fig. S6 *D* and *E*). Analysis of TH content, both by immunofluorescence densitometry (Fig. 5*A*) and Western blotting (*SI Appendix*, Fig. S6*F*), did not reveal any difference between CTR and *Igf1* cKO mice. TH activity is regulated by phosphorylation in vivo, and depolarization-induced phosphorylation of residue Ser40 correlates with higher synthetic activity (40). Two weeks after *Igf1* conditional deletion, we performed densitometry of phospho Ser40 TH and found that *Igf1* cKO mice show reduced levels of phosphorylated TH (Fig. 5B), suggesting reduced ability to synthesize DA. In agreement with this finding, we measured DA content by HPLC and found that *Igf1* cKO mice display reduced DA content in the striatum (Fig. 5*C*). These data show that absence of DA neuron-derived IGF-1 in vivo causes deficits in striatal DA content.

### IGF-1 Modulates mDA Neurons’ Firing and *Igf1* cKO mDA Neurons Show Aberrant Firing.

**Fig. 6.**
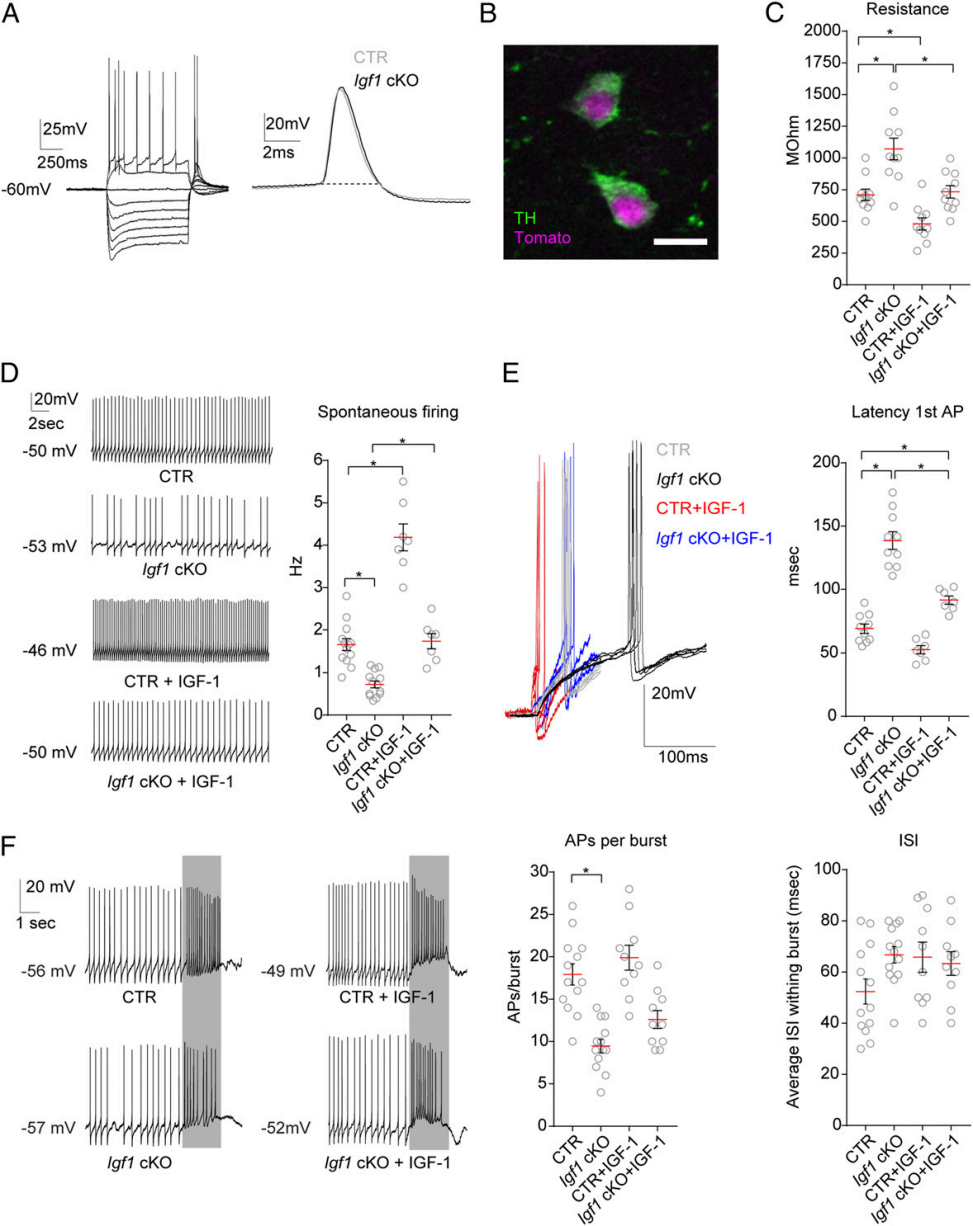
IGF-1 modulates SNc DA neuron firing properties and *Igf1* cKO SNc DA neurons display impaired autonomous and burst-like firing. SNc mDA neurons were identified by their sag in response to hyperpolarizing current injection, broad action potentials (>2 ms) (*A*), slow spontaneous firing (<4 Hz) (*D*), and expression of reporter fluorescence protein tdTomato (*B*). Representative image in *B* shows immunofluorescence for TH on a PFA-fixed brain slice, after patching. TH immunofluorescence colocalizes with *Slc6a3*^*CreERT2/+*^- driven tdTomato expression. (Scale bar: 20 μm.) (*C*) IGF-1 treatment (50 ng/mL) decreased membrane resistance (MOhm) in CTR neurons. *Igf1* cKO mDA neurons showed increased membrane resistance, compared with CTR, and 50 ng/mL IGF-1 treatment rescued membrane resistance values of *Igf1* cKO mDA neurons [*P* CTR vs. *Igf1* cKO = 0.0007, *P* CTR vs. CTR + IGF-1 = 0.0453, *P Igf1* cKO vs. *Igf1* cKO + IGF-1 = 0.0015; *F*_(3,__36)_ = 16.87]. (*D*) Shows representative spontaneous firing recorded from SNc DA neurons. *Igf1* cKO neurons showed slower firing frequency (hertz) compared with CTR neurons. IGF-1 treatment (50 ng/mL) caused an increase in the frequency of spontaneous firing in CTR neurons and rescued the *Igf1* cKO neurons’ phenotype [*P* CTR vs. *Igf1* cKO = 0.0003, *P* CTR vs. CTR + IGF-1 < 0.0001, *P Igf1* cKO vs. *Igf1* cKO + IGF-1 = 0.0010; *F*_(3,__36)_ = 65.51]. (*E*) *Igf1* cKO mDA neurons showed longer latency to trigger action potentials (milliseconds), compared with CTR. IGF-1 application (50 ng/mL) partially rescued *Igf1* cKO neuron’s phenotype [*P* CTR vs. *Igf1* cKO < 0.0001, *P Igf1* cKO vs. *Igf1* cKO + IGF-1 < 0.0001, *P* CTR vs. *Igf1* cKO + IGF-1 = 0.0195; *F*_(3,__30)_ = 59.26]. (*F*) Traces show representative bursts of action potentials induced by local stimulation (gray bars). *Igf1* cKO mice showed fewer action potentials (APs) per burst and a trend toward higher interspike interval within the burst, compared with CTR neurons [APs per burst: *P* CTR vs. *Igf1* cKO < 0.0001; *F*_(3,__42)_ = 17.75]. Graphs show means ± SEM, together with individual values pooled from *n* = 3 mice for CTR and *Igf1* cKO, *n* = 5 mice for CTR + IGF-1, and *n* = 4 mice for *Igf1* cKO + IGF-1. Neurons responsive to 50 ng/mL IGF-1 treatments are plotted in the CTR + IGF-1 and *Igf1* cKO + IGF-1 groups. Groups were compared with one-way ANOVA with Tukey’s correction for multiple comparisons. *P < 0.05.

IGF-1 has been shown to modulate the properties of ion channels and neuronal excitability (41, 42), and we found that IGF-1 application on mDA neuron decreased the amount of DA released at the level of the cell body (Fig. 3). Since DA is an inhibitor of mDA neuron firing (23), we hypothesized that IGF-1-mediated suppression of DA release might promote mDA neuron activity and that, conversely, the increase in DA release from the cell body, in the absence of neuronal IGF-1 in *Igf1* cKO mice, might interfere with DA neuronal activity. To explore the effect of DA neuron-derived IGF-1 on the electrophysiological properties of mDA neurons, we used an acute preparation of ventral midbrain slices obtained from CTR or *Igf1* cKO mice, expressing reporter fluorescent protein tdTomato in mDA neurons, 2 to 4 weeks after IGF-1 conditional deletion. We also treated CTR and *Igf1* cKO brain slices with IGF-1, to analyze the acute effect of IGF-1 on mDA neuron firing. mDA neurons were identified by their anatomical location in the SNc, their spontaneous firing, broad action potentials, the presence of a sag in membrane potential in response to a hyperpolarizing current injection (43) (Fig. 6 *A* and *D*), and the expression of tdTomato (Fig. 6*B*). We found that mDA neurons from *Igf1* cKO mice showed both passive and active changes in membrane properties. Membrane resistance was increased (Fig. 6*C*), and since cell body size of *Igf1* cKO neurons was not affected (*SI Appendix*, Fig. S6*E*), these data suggest changes of intrinsic membrane excitability. mDA neurons generate spontaneous action potentials in ex vivo preparations (43) and deletion of IGF-1 in *Igf1* cKO mDA neurons resulted in a markedly slower firing frequency, compared with CTR neurons (Fig. 6*D*). To determine the effect of acute application of IGF-1 on mDA neuron firing we treated both CTR and *Igf1* cKO mDA neurons with 50 ng/mL IGF-1. We found that IGF-1 increased the spontaneous firing frequency of 43.75% of CTR mDA neurons (7 out of 16 neurons, from five different mice). In addition, IGF-1 treatment rescued the firing deficit in 63.63% of *Igf1* cKO (7 out of 11 neurons, from four different mice) mDA neurons (Fig. 6*D*) (CTR vs. *Igf1* cKO percentage of responders not significant; *P* = 0.440; Fisher’s exact test). The time interval for CTR and *Igf1* cKO mDA neurons to increase firing frequency after IGF-1 treatment, was 6 ± 1 min and 7 ± 1 min, respectively (*n* = 7 for both groups, mean ± SEM). mDA neurons in vivo can fire action potentials in bursts, which are thought to increase DA release and facilitate the engagement of goal-orientated behaviors (44). Burst-like firing can be induced in ex vivo preparations by stimulating local glutamatergic afferents (43). Following electrode stimulation, we calculated the latency to trigger the first action potential. *Igf1* cKO preparations had a longer latency to fire the first action potential (Fig. 6*E*). Interestingly, 50 ng/mL acute application of IGF-1 partially rescued the deficit in *Igf1* cKO mDA neurons, by robustly reducing the latency to fire action potentials following electrical stimulation (Fig. 6*E*). We also quantified the number of action potential per burst in CTR and *Igf1* cKO mDA neurons. We determined that mutant neurons showed reduced capability to trigger bursts of firing, with fewer action potentials per burst and a trend toward longer interspike intervals compared with CTR neurons (Fig. 6*F*); 50 ng/mL IGF-1 treatment did not rescue the number of action potentials per burst in *Igf1* cKO neurons.

Overall these results show that in the absence of mDA neuron-derived IGF-1 production, mDA neurons are less excitable and display severe deficits in both spontaneous and burst-like firing, thus establishing that DA neuron-derived IGF-1 plays a role in shaping DA neuronal activity. Importantly, acute ectopic IGF-1 application determines a robust increase in mDA neuron spontaneous firing and is sufficient to rescue the spontaneous firing deficit in *Igf1* cKO neurons.

In addition to electrophysiological properties, we have assessed through fast-scan cyclic voltammetry (FCV) at carbon fiber microelectrodes whether *Igf1* cKO mice showed changes in the ability to release DA in the striatum. Mean peak of [DA]o evoked by either single pulses (1p; *SI Appendix*, Fig. S8 *A* and *B*) or trains of pulses (4p, 100 Hz; *SI Appendix*, Fig. S8 *C* and *D*) were not significantly different between *Igf1* cKO and CTR animals, in either dorsal or ventral striatum. These results suggest that the ability to release DA in the striatum is not compromised in *Igf1* cKO mice.

### *Igf1* cKO Mice Display Deficits in DA-Controlled Behaviors.

**Fig. 7.**
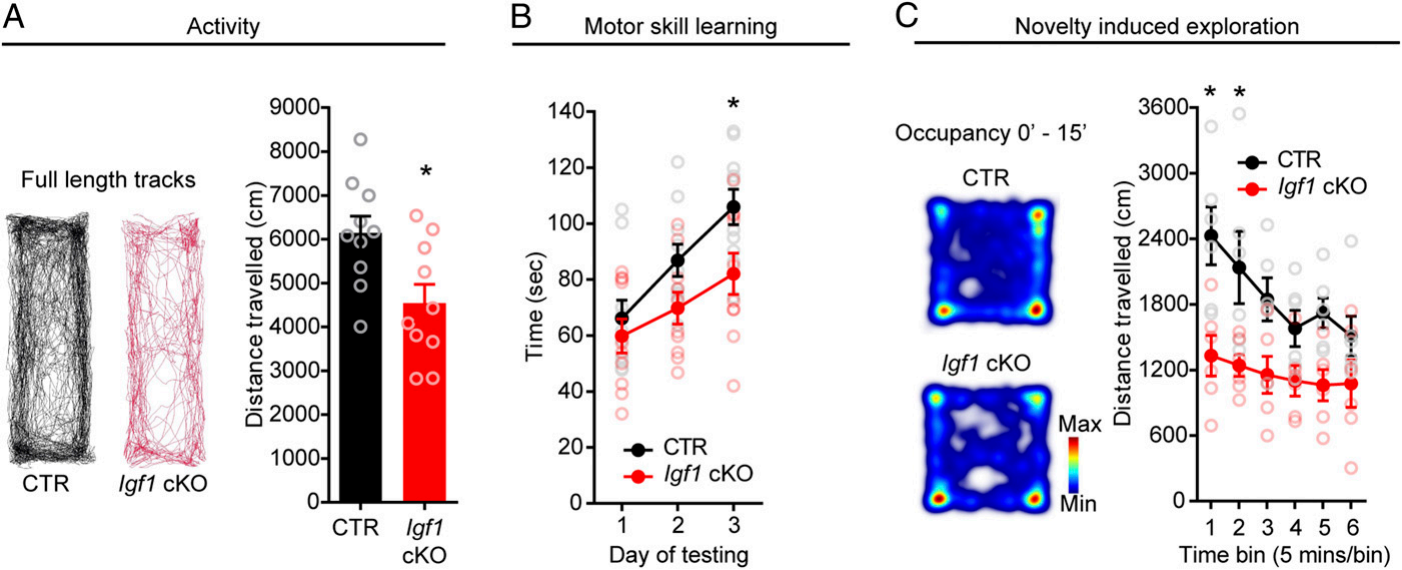
*Igf1* cKO mice show less spontaneous locomotion, impaired motor skill learning, and reduced novelty-induced exploration. (*A*) Representative cumulative tracks of mice in their home cage over 30 min. *Igf1* cKO mice show less spontaneous movement (distance traveled, centimeters), compared with the CTRs (*n* = 10, nine males and one female per genotype. *P* = 0.0123, *t* = 2.784 df = 18; two-tailed unpaired Student’s *t* test). (*B*) Mice were tested on accelerating rotarod, over 3 days, and latency to fall (time, seconds) was scored. *Igf1* cKO mice show impaired learning [*n* = 10, nine males and one female per genotype. *P* CTR vs. *Igf1* cKO day 3 = 0.0310, day 2 = 0.1888, day 1 = >0.9999; *F*_(2,__36)_ = 3.477; two-way ANOVA with Bonferroni’s correction for multiple comparisons]. (*C*) Panel on the left shows examples of raw data from individual mice during the open-field test, as calculated between 0 min and 15 min during the task. Blue to red indicates low to high occupancy. Distance traveled (centimeters) has been calculated for 5-min time bins over a total of 30 min; *Igf1* cKO mice showed reduced novelty-induced exploration compared with CTRs [*n* = 6; five males and one female per genotype. *P* CTR vs. *Igf1* cKO time bin 1 = 0.0012, time bin 2 = 0.0115, time bins 3, 4, 5, and 6, *P* = >0.05; *F*_(5,__50)_ = 1.928; CTR, *P* time bin 1 vs. time bin 6 ≤0.0001; *Igf1* cKO, *P* time bin 1 vs. time bin 6 = 0.7171; *F*_(5,__50)_ = 1.928; two-way ANOVA with Sidak’s correction for multiple comparisons]. Graphs show mean ± SEM. *P < 0.05.

Since *Igf1* cKO mice showed reduced DA content and reduced activity of mDA neurons, we then determined whether these defects might result in abnormal DA-dependent behaviors in these mice. We therefore assessed the performance of *Igf1* cKO and CTR mice in various DA-dependent behavioral assays, 2 to 4 weeks after *Igf1* conditional deletion. DA-deficient mice have been shown to be hypoactive (45), and slowness or lack of movement is a cardinal motor symptom of Parkinson’s disease that is associated with reduced DA transmission (46). We measured the spontaneous locomotion of mice by using a home-cage activity test. *Igf1* cKO mice traveled smaller distances than CTR mice therefore revealing reduced spontaneous locomotor activity (Fig. 7*A*). Efficient DA transmission is also important to regulate motor coordination and acquisition of new motor skills (47, 48). To address the effect of IGF-1 loss on such behaviors, we examined the performances of mice on an accelerating rotarod. As expected, CTR mice increased the length of time they remained on the rotarod, demonstrating motor skill learning over time (Fig. 7*B*). In contrast, *Igf1* cKO mice showed reduced levels of improvement. Interestingly, CTR and cKO mice did not present differences on the first day of testing, suggesting that loss of *Igf1* cKO does not affect motor coordination per se but learning of a new motor skill (Fig. 7*B*). mDA neurons fire bursts of action potentials during presentation of unexpected reward and novel stimuli (49), and burst firing in the SNc has been shown to be important for mice to engage in exploratory behavior of novel environments (50). Since *Igf1* cKO neurons present a deficit in burst-like firing, we asked whether this resulted in impaired exploratory behavior of *Igf1* cKO mice. In an open field test, CTR mice showed novelty-driven exploratory behavior of the environment which declined over time. In marked contrast, *Igf1* cKO mice showed decreased exploratory behavior and moved within the novel environment at a constant rate over time (Fig. 7*C*). To determine whether the exploratory behavior resulted in part from increased stress or anxiety, we compared how much time CTR and *Igf1* cKO mice spent in the more anxiogenic center of the arena vs. its less anxiogenic border. We also performed a light–dark box test to assess anxiety-like behavior. Both tests did not reveal any difference between CTR and *Igf1* cKO mice, indicating that the reduced exploratory behavior of *Igf1* cKO mice does not reflect a difference in anxiety-like behaviors (*SI Appendix*, Fig. S9 *A* and *B*). We also did not find differences in the forced swim assay (to test depression-like states) (*SI Appendix*, Fig. S9*C*), sucrose preference test (to test anhedonia) (*SI Appendix*, Fig. S9*D*), or novel object recognition test (*SI Appendix*, Fig. S9*E*). Together, our analyses demonstrate that IGF-1 production by mDA neurons is essential for the maintenance of several but not all DA-regulated behaviors.

## Discussion

We show with this study that mDA neurons synthesize IGF-1 and release it in an activity-dependent manner, and that IGF-1 is a critical regulator of mDA neuron firing and mDA-mediated behaviors.

We found that mDA neurons express both IGF-1 transcripts and protein. Of note, according to the human Allen Brain Atlas (human.brain-map.org/), *Igf1* transcript is also expressed in the midbrain of healthy human samples, raising the possibility that IGF-1 may also have a role in mDA neurons in humans. To date, several proteins have been found to be differentially expressed between SNc and VTA and are thought to establish functional differences and susceptibility to neurodegeneration between these two groups (51). We found, in agreement with previous reports (13, 14), that *Igf1* transcripts and protein were enriched in SNc neurons. In line with a potential greater influence of IGF-1 on SNc neurons, we found that behavioral tasks designed to assess anxiety, depression, and anhedonia, usually associated with the mesolimbic pathway that is predominantly regulated by VTA neurons, were not affected in *Igf1* cKO mice. We show also that IGF-1 is localized at the level of the cell body in mDA neurons and is not detected in striatal DA axons. A previous study showed that insulin signaling, but not IGF-1R signaling, is involved in regulating DA release from DA axons, via cholinergic interneurons in the striatum, supporting the notion that IGF-1 is not engaged in regulation of DA signaling at the level of DA axons (52). We therefore propose that DA neuron-derived IGF-1 mainly acts at the level of the somatodendritic compartment to regulate mDA neurons in an autocrine/paracrine fashion.

In the nervous system, neuropeptides form a very heterogeneous class of molecules that are usually secreted in response to neuronal activation and change the properties of classic neurotransmitter transmission (19, 20). We found indirect evidence that mDA neuron-derived IGF-1 is secreted upon increase in mDA neuron activation. IGF-1 has been shown to be secreted from olfactory bulb neurons following intense stimulation (10, 53), suggesting that its mode of release in the CNS is conserved across different neuronal cell types. Interestingly, IGF-1 secretion in basal conditions is likely to be limited since it is unchanged when DA neuron activity is decreased or exocytosis is blocked.

Circulating hormones control mDA neuron firing (16, 17), and at present we do not know whether serum-derived IGF-1 contributes to mDA neuron modulation. Nevertheless, we show that deletion of DA neuron-derived IGF-1 is sufficient to cause robust functional and behavioral deficits. Our *Igf1* cKO mice are hypodopaminergic. TH is the rate-limiting enzyme in the synthesis of DA and its phosphorylation at Ser40 increases its activity both in vitro and in vivo (40). We found that lack of IGF-1 signaling leads to a decrease in TH phosphorylation without affecting total TH level. It has been reported that IGF-1 increases TH activation in chromaffin cells (39) and that Ser40 can be phosphorylated by PKC (40), which is an intracellular effector of IGF-1 (54). Also, depolarization increases TH phosphorylation (55). Both the reduction of IGF-1 signaling and reduced firing rate may thus contribute to the reduced activity of TH, leading to reduced synthesis of DA in the midbrain and consequently less DA being transported to the striatum in *Igf1 cKO* mice.

We found that exposure of mDA neurons to IGF-1 reduces the rate of DA release from their cell bodies. We have also shown that the electrophysiological properties of mDA neurons and their regulated behaviors were strongly impaired after DA neuron-derived IGF-1 deletion. Absence of neuronal IGF-1 may modulate DA transmission by both regulating DA release from the cell body and by interfering with the intrinsic electrophysiological properties of DA neurons. mDA neurons are atypical because they release DA not only from axons but also from the somatodendritic compartment. Somatodendritic DA release imposes an autoinhibitory pressure on mDA neuron firing via the D2R and also reduces DA release at the striatum (29, 30, 56). Although the molecular machinery responsible for DA release from the somatodendritic compartment has not been fully elucidated, it is thought to be exocytotic, Na^+^- and Ca^2+^-dependent, and to be mediated by a different cohort of proteins, compared with those regulating axonal DA release [e.g., synaptosomal-associated protein (SNAP)/vesicle-associated membrane protein (VAMP)/syntaxin] (29, 30). IGF-1 is a pleiotropic factor and at present we do not know the downstream effectors of IGF-1 signaling that mediate reduced DA release. IGF-1 signaling has previously been linked to proteins that are involved in exocytosis and neurotransmitter release (57, 58). We could speculate that engagement of IGF-1 signaling may lead to rapid posttranslational modification of DA exocytotic machinery, resulting in a decrease in the rate of DA release from the somatodendritic compartment. Regardless of the exact mechanism behind IGF-1-mediated decrease of DA release, the use of FFN102 allowed us to determine the functional effect of IGF-1 on DA release in an intact ventral midbrain environment. This result demonstrates that IGF-1 promotes a strong functional response by slowing down net DA release, presumably resulting in a reduction of DA-mediated autoinhibition through D2R receptors.

The analysis of *Igf1* cKO mice showed that depletion of IGF-1 signaling in vivo has a severe impact on DA neuron firing, and importantly on shaping behaviors known to engage DA transmission. Electrophysiological analysis demonstrated that *Igf1* cKO mDA neurons have robust deficits in autonomous and burst-like firing. Characterization of the mechanism(s) through which IGF-1 dampens mDA neuron firing is beyond the scope of this study. Nevertheless, we can speculate that both cell- and non-cell-autonomous mechanisms are involved. Molecular determinants of tonic firing include persistent subthreshold sodium current (59), L-type calcium currents (60), and the hyperpolarization-activated cation current I_H_ (61). However, burst firing is heavily dependent on NMDA receptor (NMDAR) (62) and synaptic inputs (63). Engagement of IGF-1 intracellular pathways has already been linked to the modulation of these determinants in other cells. For example, Src kinases [downstream of IGF-1R signaling (64)] modulate HCN channel gating properties (65), IGF-1 potentiates the activity of CaV1.3 channels in cortical neurons (66), and IGF-1 robustly increases surface expression of NMDAR in cerebellar granule cells by AKT-dependent phosphorylation (67). Interestingly, acute IGF-1 treatment strongly modulates spontaneous firing and rescues the *Igf1* cKO neurons phenotype, showing that IGF-1 regulates spontaneous activity of DA neurons. In addition, IGF-1 effects on spontaneous firing were detectable after a few minutes from its application, suggesting that IGF-1 tunes spontaneous firing by modulating the localization and/or activity of ion channels (or their modulators), rather than their de novo expression. We also found that acute IGF-1 treatment does not rescue the burst-firing deficit observed in *Igf1* cKO neurons. Burst firing of mDA neurons is dependent on presynaptic input (63). We could speculate that, in addition to the defects in intrinsic firing properties of mDA neurons, lack of DA neuron-derived IGF-1 may also alter the presynaptic input on mDA neurons over time in vivo, and hence influence their firing properties. Therefore, we hypothesize that a longer IGF-1 treatment (e.g., overexpression in mDA neurons or chronic IGF-1 delivery by cannulation) may rescue the *Igf1* cKO burst-firing deficit. Of note, VIP cortical neuron-derived IGF-1 has recently been reported to modulate the number and/or strength of functionally inhibitory synapses onto these neurons and in vivo overexpression of IGF-1 in VIP neurons selectively promotes inhibition onto VIP neurons (12). Interestingly, we found that striatal release of DA, assessed through FCV, was not affected in *Igf1* cKO mice. This finding strongly suggests that the deficits observed in *Igf1* cKO mice are due to changes in DA neuron firing properties and somatodendritic functions, rather than reduced ability to release DA in the striatum.

Lack of mDA neuron-derived IGF-1 severely impaired the performance of mice in behavioral assays dependent on DA transmission, emphasizing the importance of the neuromodulatory effect of IGF-1 on mDA neuron activity. Reduced DA transmission has been associated with hypoactivity in mice (45). In humans, reduced DA transmission as a result of SNc neuron degeneration is a hallmark of Parkinson’s disease (4). The clinical manifestations include bradykinesia/akinesia (46) and reduced efficiency of motor learning (68), and impaired motor learning has been proposed as a presymptomatic stage of Parkinson’s disease (48). In our studies, we have shown that *Igf1* cKO mice are impaired in behavioral paradigms that are known to be influenced by DA neuronal transmission. *Igf1* cKO mice showed overall hypokinesia and did not acquire new motor skills as efficiently as CTR mice. DA phasic signaling has been linked to the processing of novel stimuli. In particular, absence of burst firing in a subpopulation of SNc neurons has been shown to be sufficient to impede novelty-induced exploration (50). *Igf1* cKO mice have reduced DA levels and reduced spontaneous and burst-like firing of mDA neurons and present a drastic reduction of exploration in a novel environment. Importantly, liver-derived *Igf1* cKO mice do not show impairments in exploratory activity, indicating that exploratory behavior is modulated specifically by neuronally derived and not by systemic circulating IGF-1 (69).

We suggest that IGF-1 is released from mDA neurons during behavioral paradigms when sustained or increased DA transmission is required. The activity-dependent release of IGF-1 at the level of cell body and the resultant reduction in D2R-mediated autoinhibition may promote a quick adaptation of mDA neurons to enable more intense and sustained firing. We also hypothesize that reduction of DA neuron-derived IGF-1 signaling in vivo leads to a reduction in both tonic and phasic DA neuron firing, that together with reduced DA synthesis, cause impairments in the execution of behaviors that require efficient DA transmission.

In conclusion, by showing effects on DA neuron firing and DA-mediated behaviors, our findings have demonstrated a previously unrecognized and physiologically relevant neuroregulatory role for DA neuron-derived IGF-1.

## Materials and Methods

### Mice and Tamoxifen-Induced Recombination.

Research involving animals has been approved by the Home Office–Secretary of State (ref. no. 80/2550) and Institutional Animal Welfare and Ethical Review Body of The Francis Crick Institute, which has been approved by the Home Office as a breeding, supplying, and research establishment (ref. no. 70/9092). All procedures were carried out in accordance with the Animals (Scientific Procedure) Act of 1986. Mice were maintained on a 12-h light/dark cycle with water and food for ad libitum consumption. *Slc6a3*^*CreERT2/+*^ (70) and *Igf1*^*flox/flox*^ (71) mice were crossed to produce *Slc6a3*^*CreERT2/+*^; *Igf1*^*flox/flox*^ mice. Cre-mediated recombination of *Igf1* alleles in DAT expressing neurons was achieved by i.p. injections of tamoxifen at 8 to 12 weeks of age. Tamoxifen (Sigma) was reconstituted in 10% ethanol/90% corn oil (Sigma) at a concentration of 10 mg/mL and kept refrigerated and protected from light. All mice were injected with 1 mg of tamoxifen twice a day, for five consecutive days. *Slc6a3*^*CreERT2/+*^; *Igf1*^*flox/flox*^ mice that were tamoxifen-injected are referred to as *Igf1* cKO, while mice harboring only the floxed allele (*Igf1*^*flox/flox*^) were used as controls, unless otherwise stated. Mice were analyzed 2 to 4 weeks after tamoxifen injections. We have not observed any gross effect on body weight upon *Igf1* recombination. *Igf1* cKO mice were not used for breeding purposes. Both non-tamoxifen-injected *Slc6a3*^*CreERT2*^*/+*; *Igf1*^*flox/flox*^ and *Igf1*^*flox/flox*^ mice were fertile and healthy. In accordance to NC3Rs practice (https://www.nc3rs.org.uk/the-3rs), both males and females were used for this study.

### Immunofluorescence.

#### Brain sections.

Brains were fixed by transcardiac perfusion with 4% paraformaldehyde (PFA)/PBS, postfixed overnight at 4 °C, cryoprotected in 30% sucrose/PBS, and cryosectioned. For IGF-1 immunofluorescence, sections were processed to citric acid antigen retrieval prior to immunolabeling. Sections were blocked in 1% BSA and 0.2% Triton X-100 in PBS and incubated with primary antibodies overnight at 4 °C. Sections were incubated with appropriate fluorescent secondary antibodies (1:250; Invitrogen) 1 h at room temperature and mounted with Vectashield antifade mounting agent (Vector). All antibodies were diluted in 1% BSA and 0.2% Triton X-100/PBS. Primary antibodies used were anti-TH (1:1,000; Pel-Freez P40101-150 and Millipore AB1542), anti-IGF-1 (1:200, AF-291-NA; R&D Systems), anti–IGF-1R (1: 200, 9750; Cell Signaling), anti-phosphoSer40 TH (1:1,000, AB5935; Merck), anti-phosphoTyr1135/1136 IGF-1R (1:200, 3024; Cell Signaling), and anti-phosphoSer235/236 S6 Ribosomal protein (1:100, 4858; Cell Signaling).

#### Primary culture.

Cells were rinsed in PBS, fixed with 4% PFA/PBS for 10 min, and permeabilized with 0.25% Triton X-100/PBS for 10 min at room temperature. Samples were blocked with 3.5% BSA/PBS 1 h at room temperature and incubated with primary antibodies overnight at 4 °C. Cells were incubated with appropriate fluorescent secondary antibodies (1:250; Invitrogen) 1 h at room temperature and mounted with hard-setting antifade mounting agent (Aqua-Tex). All antibodies were diluted in 3.5% BSA/PBS. Primary antibodies used were anti-MAP2 (1:2,000, M4403; Sigma), anti-D2R (1:500, AB5084P; Chemicon), anti-IGF-1 (1:200, ab9572; Abcam), and anti-Fos (1:4,000, ABE457; Millipore).

### Primary Culture of mDA Neurons and Pharmacological Treatments.

Pregnant mice were killed by cervical dislocation and E16 embryos dissected out (8 to 12 embryos per preparation). Embryos were immediately placed in cold L-15 media (Gibco), the brain was exposed, and the ventral midbrain was isolated. Ventral midbrains were cut into small fragments, pooled together, and digested with papain (P3125; Sigma) for 20 min at 37 °C in a humidified incubator with 5% CO_2_ atmosphere. Following papain digestion, ventral midbrains were dissociated by gentle pipetting and spun through a BSA column. Purified cells were resuspended in complete media (Neurobasal, Gibco 21103-049 supplemented with 1:100 penicillin–streptomycin, Sigma P4333; 1:100 Glutamax, Gibco 35050038; 1:50 B27, Gibco 17504044; and 10% FBS, Sigma F9665); 150,000 cells were plated on 13-mm glass coverslips, precleaned with nitric acid and precoated with poly-l-ornithine. Media was changed every 3 days, and to block glial-cell proliferation 10 μM Ara-C was added to the media 3 days after plating. All pharmacological treatments were carried out 6 days after plating, with chemicals prepared in complete media, for 1 h at 37 °C in a humidified incubator with 5% CO_2_ atmosphere. The following compounds were used: 10 μM sulpiride (Sigma), 1 μM TTX (Tocris), 1 μM quinpirole (Sigma), and 100 μM EXO1 (Tocris and ref. 26). After treatments, cells were immediately processed for immunofluorescence (discussed above).

### Electrophysiology.

Whole-cell slice patch-clamp recordings were performed using standard techniques, as in previous work (72). Brain slices were prepared 2 to 4 weeks after tamoxifen injections from male mice. CTR (*Slc6a3*^*CreERT2/+*^; *Igf1*^*+/+*^; *Gt(ROSA)26Sort*^*m14(CAG-tdTomato)Hze/+*^) and *Igf1* cKO (*Slc6a3*^*CreERT2/+*^; *Igf1*^*flox/flox*^; *Gt(ROSA)26Sort*^*m14(CAG-tdTomato)Hze/+*^) mice were analyzed at the same time after tamoxifen-mediated recombination. Mice were quickly killed by cervical dislocation, and their brains were removed and glued onto a stage. Acute coronal slices, 200 µm thick, were cut while immersed in ice-cold ACSF slicing solution using a vibratome. Slices were then transferred to a submerged-type chamber and incubated for 30 min in ACSF at 35 °C, and then at room temperature (25 °C) until they were used for recording. Neurons were visualized in acute brain slices with an upright Olympus BX51WI microscope equipped with an oblique condenser and appropriate filters. Slices were superfused with ACSF at a rate of 4 to 5 mL/min. SNc dopaminergic neurons were identified by their anatomical location, expression of fluorescent reporter, and electrical fingerprint (*Results*). Cells were randomly sampled throughout the full anatomical extent of the SNc. To assess differences between CTR and *Igf1* cKO SNc neurons, we performed whole-cell current-clamp recordings in the presence of synaptic blockers 6-cyano-7-nitroquinoxaline-2,3-dione (CNQX, 10 µM), (2R)-amino-5-phosphonovaleric acid (D-AP5, 50 µM) and SR95531 (GABAzine, 20 µM). For the generation of burst firing, the selective metabotropic glutamate receptor type 1 (mGlu1) antagonist YM 202074 (1 µM), GABAA antagonist SR95531 (1 µM), and GABAB antagonist (CGP 52432, 1 µM) were added. For whole-cell recordings, pipettes were filled with intracellular solution containing 120 mM potassium-gluconate, 10 mM KCl, 10 mM Hepes, 0.1 mM EGTA, 4 mM K_2_ATP, 2 mM Na_2_ATP, 0.3 mM Na_2_GTP, and 2 mM MgCl_2_; pH 7.3 with KOH.

#### Generation of burst firing in SN mDA neurons.

Burst firing was generated through local electrical stimulation (50 to 100 stimuli at 50 to 100 Hz; stimulus duration, 0.1 ms; stimulus intensity, 200 to 500 µA) of glutamatergic afferents in the presence of GABAA, GABAB, and type1 metabotropic glutamate receptor antagonists to block inhibition and maximize glutamatergic excitation. Bipolar electrical stimulation was applied via a bipolar stimulating electrode (FHC).

#### Data analysis.

Data were analyzed using IgorPro 6 (Wavemetrics), Origin Pro-8.6 (OriginLab), Prism 6.0 (GraphPad). Synaptic stimulation artifacts were removed by deleting up to 1 ms of data for each artifact and then replacing the data with a straight line that spanned the deletion. Action potential initiation was detected as described previously (73). Burst firing was defined as firing of a frequency greater than the mean spontaneous firing frequency + 3 SD (43).

#### Chemicals and solutions.

All drugs were from Sigma, but IGF-1 (250-19; Peprotech). IGF-1 was delivered in ACSF at a final concentration of 50 ng/mL. ACSF and the ice-cold slicing solutions were gassed with 95% O_2_ and 5% CO_2_, and they contained the following: ACSF: 125 mM NaCl, 2.5 mM KCl, 1 mM MgCl_2_, 2 mM CaCl_2_, 1.2 mM NaH_2_PO_4_, 21 mM NaHCO_3_, and 1 mM d-(+)-glucose; slicing solution: 2.5 mM KCl, 1.3 mM NaH_2_PO, 26.0 mM NaHCO_3_, 213.3 mM sucrose, 10.0 mM d-(+)-glucose, 2.0 mM MgCl_2_, and 2.0 mM CaCl_2_.

#### Statistical analysis.

No statistical methods were used to predetermine sample sizes, but our sample sizes are similar to those reported in previous publications (43, 74).

### Behavior.

In line with NC3Rs guidelines both male and female mice were used for behavioral tests. Different cohorts of mice were used for the various behavioral assays. Mice were kept on a standard 12-h light/dark cycle (light on at 0700 hours) with standard mouse chow and water available for ad libitum consumption in a temperature- and humidity-controlled (21 ± 2 °C; 50 ± 5%) environment. Before behavioral testing, mice were single-housed for at least 1 week to minimize social interaction effects on behavior. All behavioral tests were carried out during the light phase between 1000 hours and 1600 hours, and never on the day, or the day after, cage cleaning. Tests were carried out between 2 and 4 weeks after *Igf1* conditional deletion. Before behavioral tests, mice were moved into the testing room and habituated for 1 h before starting the test. In behavioral experiments, CTR and mutant genotypes were assayed in parallel when possible, or genotypes were interleaved during trials to avoid any order effects. No mice were excluded from the analysis. See *SI Appendix* for a detailed description of the behavioral tests.

See *SI Appendix* for details on image acquisition, analysis and data normalization, ISH, FFFN102 imaging, DA HPLC, RT-qPCR, Western blotting, immunoprecipitation, and statistics.

## Supplementary Material

Supplementary File
