# Supplementary material for: Dopamine neuron-derived IGF-1 controls dopamine neuron firing, skill learning, and exploration
Source: Proc Natl Acad Sci U S A. 2019 Feb 11;116(9):3817–26. doi: 10.1073/pnas.1806820116 (PMC6397563; doi:10.1073/pnas.1806820116)
Supplement: Supplementary File [file pnas.1806820116.sapp.pdf]

PNAS
www.pnas.org

Supplementary Information for

Dopamine neuron-derived IGF-1 controls dopamine neuron firing, skill learning and exploration.

Alessandro Pristerà[1], Craig Blomeley[1], Emanuel Lopes[2], Sarah Threlfell[2], Elisa Merlini[1], Denis Burdakov[1], Stephanie Cragg[2,3], François Guillemot[1] and Siew-Lan Ang[1*].

Corresponding author Siew-Lan Ang
Email: Siew-Lan.Ang@crick.ac.uk

This PDF file includes:

Supplementary materials and methods
Supplementary Figs. S1 to S9
References for SI reference citations

Other supplementary materials for this manuscript include the following:

N.A.

www.pnas.org/cgi/doi/10.1073/pnas.1806820116

Supplementary Information Text

Materials and methods.

In situ hybridisation (ISH)

Igf1 antisense digoxigenin-labelled probe was synthesised from ventral midbrain cDNA template and sequence verified. ISH was performed by standard protocol as previously described (1) on 35 μm thick brain sections. *Igf1* antisense probe was revealed by immunolabeling with anti-digoxigenin alkaline phosphatase conjugated antibody (1:1500; Roche) followed by alkaline phosphatase mediated colorimetric reaction by using nitro blue tetrazolium chloride/5-bromo-4-chloro-3-indolyl-phosphate mix (Roche).

Image acquisition, analysis and data normalisation

Both brain sections and primary culture samples were imaged on an Axio Imager.Z2 ApoTome.2 microscope (Zeiss) equipped with an AxioCam MRm camera. For the purpose of fluorescence intensity quantification or co-localisation analysis, images were acquired in structured illumination mode to achieve optical sectioning. Signal intensity of cytoplasmic fluorescence levels was quantified by Fiji image processing package. Only mDA neurons showing the nucleus in the optic sections were included in the analysis. Between two hundred and five hundred cells per condition were used for quantification purposes. Background fluorescence was subtracted from all the readings. Control and mutant (or treated, in the case of primary culture) samples were always processed and imaged in parallel with same acquisition settings. Extra care has been taken in sampling neurons from matching regions in the SNc and VTA, between control and mutant samples. Quantification was performed blinded to the genotype/treatment. Calculated raw

fluorescent density values were normalised against matching control values, pooled together and are expressed as % change *vs*. control.

False fluorescent neurotransmitter 102 (FFN102) loading and imaging

Mice were euthanized by cervical dislocation and brains quickly dissected. 250 µm thick coronal sections, containing the ventral midbrain, were cut on a vibratome in ice-cold cutting buffer (180 mM sucrose, 10 mM NaCl, 2.5 mM KCl, 25 mM $NaHCO_3$, 0.5 mM $CaCl_2$, 7mM $MgCl_2$,1.25 mM NaH_2PO_4, 10 mM glucose, pH 7.3, oxygenated with a bubbling mixture of 95% O2, 5% CO2 vol/vol). Slices were equilibrated in oxygenated artificial cerebrospinal fluid (ACSF) (125 mM NaCl, 2.5 mM KCl, 26 mM $NaHCO_3$ 0.3 mM KH_2PO_4, 2.4 mM $CaCl_2$, 1.3 mM $MgSO_4$, 0.8 mM NaH_2PO_4, 10 mM glucose, pH 7.3) for 30 min at 32°C, followed by 30 min at room-temperature. We loaded FFN102 (Abcam; ab120866. For a full characterisation of this dye, see reference (2)) into mDA neurons by incubating the brain sections in 10 µM FFN102 diluted in oxygenated ACSF for 30 min at room temperature. Brain sections were transferred into a polydimethylsiloxane custom-made imaging chamber just prior imaging. Sections were continuously superfused with oxygenated ACSF at room temperature and held in place with a custom-made platinum harp and nylon threads. Imaging started 5-10 minutes after placing the slices in the imaging chamber; this time period allowed the slices to settle in the chamber and greatly minimised spatial displacement during imaging. Experiments were carried out on a multi-photon MPSP5 upright microscope (Leica) in a dark chamber maintained at room temperature (22-24°C). FFN102 was visualised with a HCX APO L 20.0x 1.00 N.A. water immersion objective, excited with a SpectraPhysics MaiTai MP laser at 770 nm and emitted fluorescence was collected with non scanned detector

through an aSemrock 525/50 band-pass filter. Images were captured in 8 bits at 512x512-pixel resolution, 3 frames average and a scan speed of 400 Hz. *Z* stacks, at 2.0 µm step-size, were acquired over 50 min at 10 min intervals. If *z* displacement occurred during imaging, this was manually corrected during time-point acquisition by re-focusing onto the correct focal plane. 4D acquired series were analysed on Fiji. If *x*-*y* displacement occurred during acquisition this was corrected by using was StackReg plugin with Rigid Body setting. Regions of interest (ROIs) were outlined around the cell bodies of mDA neurons loaded with FFN102. FFN102 fluorescence was measured over the time series, and each neuron fluorescence reading was expressed as % change against its own reading at baseline (reading at T0 min, 1st acquisition). Background fluorescence was subtracted from each reading at each time point, to obtain background corrected fluorescence. Equivalent optic thickness between control and treated samples were analysed for fluorescent measurement. The following treatments were used: 1 µM TTX (Tocris; 1069), 50 ng/ml Des (1-3) IGF-1 (RayBiotech; 228-10835-2) added at T20 min, 1µM Tyrphostin AG 1024/DMSO (Enzo lifescience; ALX-270-217) added at T10 min.

Fast-scan cyclic voltammetry (FCV)

On each recording day, a pair of 12 weeks old male mice consisting of one control (*Igf1*$^{flox/flox}$) and one *Igf1* cKO (*Slc6a3*$^{CreERT2/+}$; *Igf1*$^{flox/flox}$), two weeks post-tamoxifen, were sacrificed by cervical dislocation and decapitated, and their brains removed over ice. Coronal slices (300 µm) were cut using a vibratome (Leica VT 1200S) in ice-cold HEPES-buffered artificial cerebrospinal fluid (aCSF) containing: 120 mM NaCl, 5 mM KCl, 20 mM NaHCO3, 6.7 mM HEPES acid, 3.3 mM HEPES salt, 2 mM CaCl2, 2 mM MgSO4, 1.2 mM KH2PO4 and 10 mM glucose and saturated with 95% O2/5% CO2.

After a minimum of 1 hour recovery at room temperature, slices were transferred to the recording chamber and maintained at ~32 ^{o}C in carbogenated bicarbonate-buffered aCSF containing: 124 mM NaCl, 3.7 mM KCl, 26 mM NaHCO3, 2.4 mM CaCl2, 1.3 mM MgSO4, 1.3 mM KH2PO4 and 10 mM glucose. Slice superfusion flow rate was 1.5 ml/min. Extracellular concentration of dopamine ([DA]o) was monitored and quantified using fast-scan cyclic voltammetry (FCV) with 7 µm-diameter carbon-fibre microelectrodes (exposed tip length, 50–100 µm, fabricated in-house) and a Millar voltammeter (Julian Millar, Barts and The London School of Medicine and Dentistry, London, UK). The applied voltage was a triangular waveform, with a voltage range of −0.7 to +1.3 to −0.7 V versus Ag/AgCl at a scan rate of 800 V/s, with a sampling frequency of 8 Hz. Electrodes were inserted in brain slices to a depth of 100 µm. Dopamine release was evoked locally by a surface, concentric bipolar Pt/Ir electrode (25 µm diameter; FHC) placed ~ 150 µm away from the carbon fibre. Stimulus pulses were generated out-of-phase with FCV scans and were applied at the lowest current that generated maximal DA release with a single stimulus pulse in wild-type animals (600 µA, 200 µs pulse duration). In each slice, [DA]o was monitored in 3 dorsal striatal sites (dorsomedial, dorso-mid, and dorsolateral) and 3 ventral striatal sites (ventromedial caudate-putamen, nucleus accumbens core, and nucleus accumbens shell). Stimuli were single pulses and 4 pulses at 100 Hz at 2.5 min intervals. Recordings in each genotype were matched each recording day for days post-tamoxifen and striatal sub-region. The order of preparation and sampling of each genotype was randomised. The evoked currents signal were confirmed as DA by comparing the peak potentials for oxidation and reduction currents with those of DA in calibration media (+500-600 and -200 mV vs.

Ag/AgCl, respectively). Electrodes were calibrated in 2 µM DA following experiments. Data reported are obtained in 6 animals per genotype. All data are expressed as means ± SEM where N is the number of animals. Comparisons for differences in means were assessed by paired Student t-tests using GraphPad Prism 6.0 (GraphPad Software, San Diego, CA, USA).

DA HPLC

Brains were rapidly dissected following cervical dislocation and decapitation. 2 mm coronal slices containing the striatum were collected and the cortex removed under a stereomicroscope. Striatal samples were weighed and snap frozen on dry ice in 200 µl 0.1 M $HClO_4$. On the day of analysis samples were thawed, homogenized and centrifuged at 16, 000 g for 15 min at 4°C. The supernatant was analysed for DA content using HPLC with electrochemical detection. Analytes were separated using a 4.6 x 150 mm Microsorb C18 reverse-phase column (Agilent) and detected using a Decade II SDS electrochemical detector with a Glassy carbon working electrode (Antec Leyden) set at +0.7 V with respect to a Ag/AgCl reference electrode. The mobile phase consisted of 13% methanol (v/v), 0.12 M NaH_2PO_4, 0.5 mM OSA, 0.8 mM EDTA, pH (4.8), and the flow rate was fixed at 1 ml/min. Analyte measurements were normalised to tissue chunk weight (pmol/mg).

RT-qPCR

RT-qPCR was quantified as previously described (3). Primer sequences for IGF-1 were F 5'-AGCTGTGATCTGAGGAGACT-3', R 5'-CACTTCCTCTACTTGTGTTC-3'. IGF-1 C_t values are normalised against GAPDH.

Western blotting and Immunoprecipitation

Mice were euthanized by cervical dislocation and ventral midbrains or striata coronal sections were dissected with brain slicer (Agar scientific). Cortices and dorsal midbrain were removed and tissues were processed for western blotting or immunoprecipitation. Western blotting was performed as previously described (3). For immunoprecipitation, striata were homogenised in lysis buffer (Pierce, 87788) supplemented with protease and phosphatase inhibitors; IGF-1R was precipitated from the cleared lysate, by incubation with anti IGF1-R (1: 100; Cell Signaling 9750) for 2 hrs at 4°C, followed by precipitation with Protein G Agarose beads for 2 hrs at 4°C. Rabbit IgG was used in parallel as a negative control. Immunoprecipitated IGF1-R and phosphorylation levels were assayed by western blotting, by probing same amount of samples with anti IGF1-R (1: 1000; Cell Signaling 9750) and anti phospho-tyrosine (1:1000; 05-321 Millipore) antibodies. Bands intensity was measured with Fiji.

Behaviour

Homecage activity: spontaneous activity in the homecage was continuously recorded by an overhead camera for a continuous period of 30 min. Mice were subsequently tracked and track length quantified in an automated fashion using Ethovision XT software version 11 (Noldus). *Open field:* exploratory behaviour was tested in an open arena made of opaque Plexiglas (50 x 50 cm square floor area). Mice were placed in the centre of the arena and recorded with an overhead camera for 30 min. Track length was automatically calculated by Ethovision XT software. Heat maps (showing occupancy) for figure preparation were generated with the “cumulated” track setting. *Light/dark box test*: for the anxiety test a dark/light box was used, composed of two chambers (dark arena 28 x

12 cm; light arena 28 x 28 cm); Mice were placed in the centre of the dark arena and their movement recorded for 15 min. Time spent between the light and dark arena was manually scored. *Motor skill learning*: motor skill learning/motor coordination was tested on an accelerating Rotarod (4 to 40 rpm over 5 min) and latency to fall was recorded, with increasing latencies indicative of better performance. Mice were tested on three consecutive days, three times a day. The daily performance of each individual is calculated as the average of the three trials. *Sucrose preference test:* anhedonia was measured using the sucrose preference task. Mice were habituated to a two bottles paradigm in their home cage over 24 h with both bottles containing water. Twenty-four hrs later, mice were given a free choice between two bottles, one with 2% (or 0.2%) (wt/vol) sucrose solution and another with tap water for 48 h (the location of the sucrose and water bottles was switched after 24 h to avoid any location preference). Extra care was taken to avoid liquid spillage, and bottles were filled in advance and kept overnight in the housing room to reduce any effects of the room temperature and pressure on the bottles. The intake of water and 2% (or 0.2%) sucrose solution and total intake was estimated by weighing the bottles before and after the test session, every 24 h. The preference for sucrose was calculated as the percentage of the sucrose solution consumed out of the total amount of liquid consumed (sucrose solution intake/total intake × 100). *Forced swim test*: cylindrical glass water tanks (height x diameter, 27 cm x 17 cm) was filled to ¾ of its capacity with water and left overnight to equilibrate to room temperature. Mice were placed on the surface of the water and their activity recorded with a camera placed on the side of the water tanks for 6 min, and the last 4 min used for analysis. Time spent moving (Motility, as defined as all movements that produced drifts

but movements for staying afloat/balancing) was manually scored. Immobility time was calculated as four minutes minus time spend moving. *Novel object recognition test*: Mice were habituated to an empty square arena (39 cm x 39 cm) on day 1. On day 2, mice were presented to two identical objects (familiar objects) for 5 min in the arena. On day 3, novel object exploration was measured during a 5 min test in the square arena as time spent exploring the novel object, which was presented together with the familiar object. Exploration was measured as the time spent by the mouse directing the nose toward the objects and touching the objects. Preference for the novel object was calculated as the percentage of total exploration time that was spent exploring the novel object. To avoid place and object preference, relative placement and choice of the novel object was randomised.

The experimenter was blind to the genotypes of the animals both during the behavioural tests and subsequent scoring of the recorded behaviours.

Statistics

Data were analysed and graphs generated with GraphPad 7.02 (Prism). Graphs are presented as means ± SEM (unless stated otherwise), together with individual data samples. Normal data distribution was assumed before performing statistical tests. Comparisons for differences in means were assessed by one-way or two-way ANOVA followed by post hoc tests or unpaired *t* tests using GraphPad 7.02. A P value of < 0.05 was used as the criterion for determining statistically significant differences. Exact P values for each comparison are provided in figure legends.

Supplementary results and figure legends

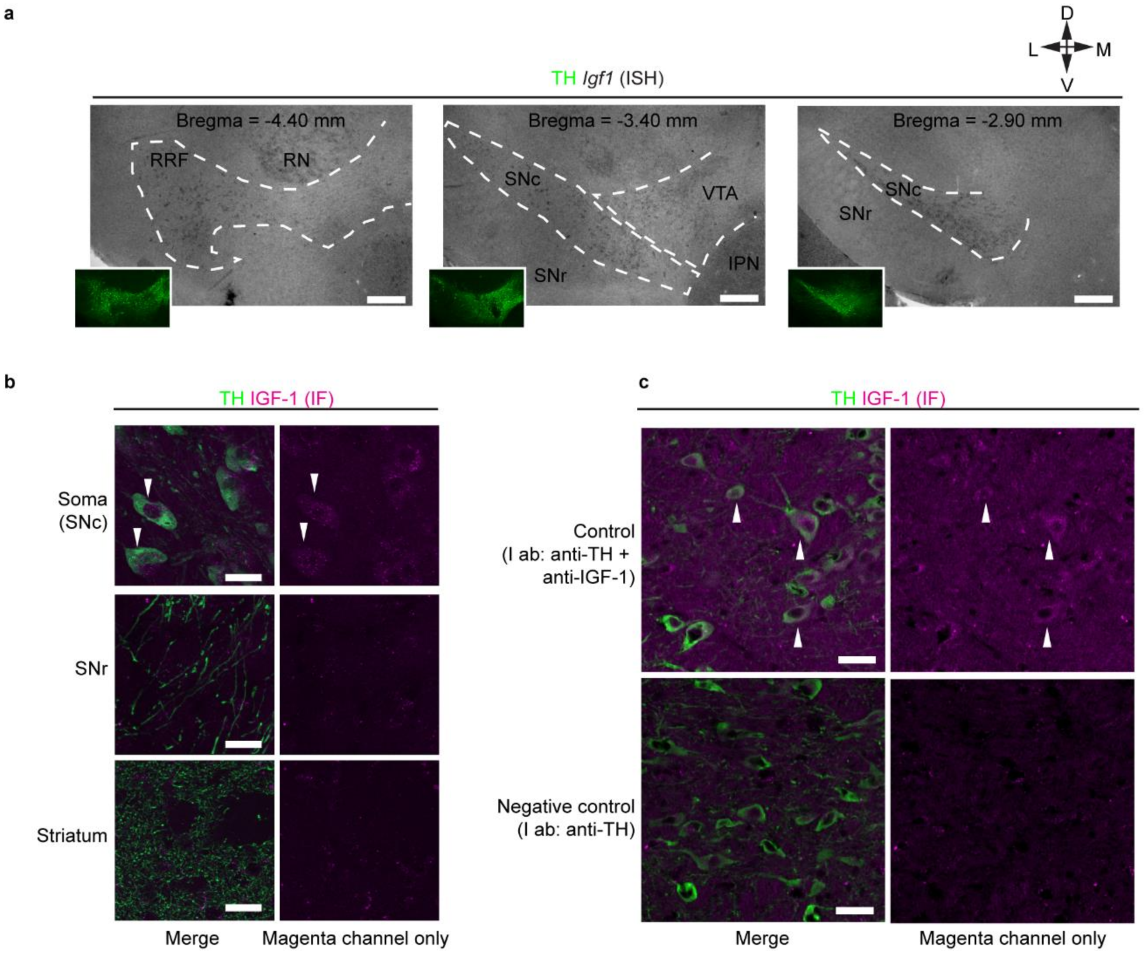

Fig. S1 *Igf1* transcript and protein spatial distribution.

(**a**) *Igf1* transcripts, detected by ISH, are distributed along the anterior-posterior axis (Bregma according to reference atlas (4)) of the ventral midbrain. Insets show TH immunofluorescence of the same region depicted in the ISH panels, to show the region occupied by mDA neurons (dotted line in the ISH panels). Crossed arrows show image orientation, with D=dorsal, V=ventral, M=medial, L=lateral. RN= red nucleus, RRF=retrorubral field, IPN=interpeduncular nucleus, VTA=ventral tegmental area, SNc=substantia nigra pars compacta, SNr=substantia nigra pars reticulata. Scale bars are 200 μm (**b**) IGF-1 protein is located in the cell bodies of TH positive neurons (Soma, SNc region), but not in dendrites (SNr) or axons (Striatum). Pictures are high

magnification and represent maximum intensity projections of a *z* stack to maximise the signal arising from TH and IGF-1 immunofluorescence signals and to minimise false negative data due to insufficient sampling. Arrowheads show TH neurons positive for IGF-1 immunoreactivity. Scale bars are 20 μm. (**c**) Panel shows the absence of IGF-1 immunoreactivity in samples processed for immunofluorescence where the primary anti-IGF-1 antibody was omitted (Negative control). IGF-1 immunoreactivity is present in the soma of TH positive neurons (arrowheads). Images showing the magenta channels' fluorescence are represented for clarity. Scale bars are 20 μm.

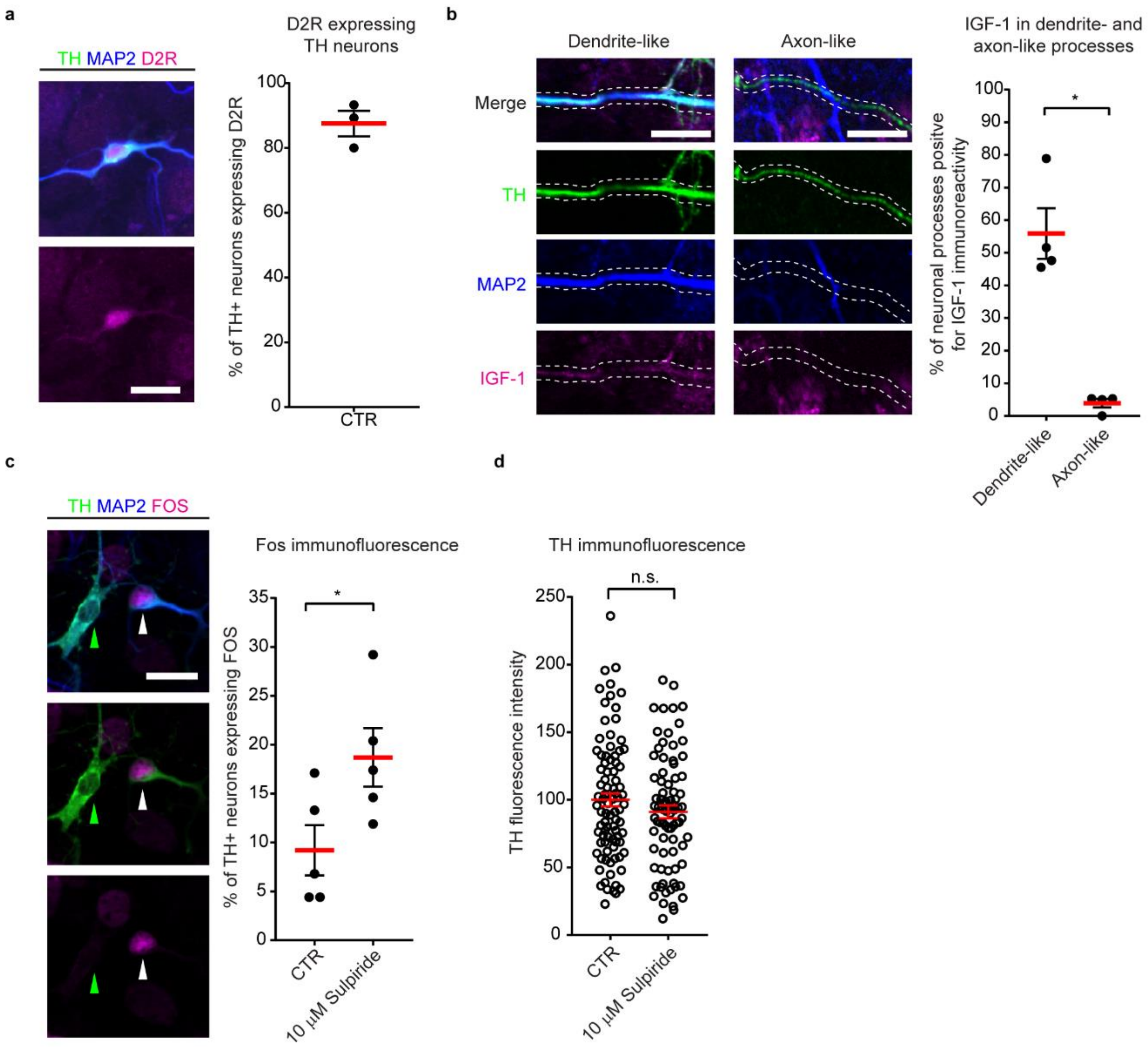

Fig. S2 Characterisation of mouse DA neuron primary culture and pharmacological stimulation.

(**a**) Image shows immunofluorescence of representative cultured mDA neuron (TH-, MAP2-positive) expressing D2R. Graphs shows the quantification of the % of TH-, MAP2-positive neurons, positive for D2R immunofluorescence (N=3 cultures, mean ± SEM). Scale bar is 20 μm. (**b**) IGF-1 does not co-localise with axon-like processes *in*

vitro. Panel show high magnification immunofluorescence of dopaminergic processes (TH-positive) *in vitro* with dendrite-like (TH-positive and MAP2 positive) and axon-like (TH-positive and MAP2 negative) features. The % of neuronal processes containing IGF-1 is quantified in the graph (mean ± SEM, N=4, P=0.0006, t=6.6 df=6; two-tailed unpaired Student's t test). Scale bar is 10 μm. (**c**) Image shows representative mDA neurons (TH-, MAP2-positive) expressing Fos (white arrowhead). Green arrowhead shows an mDA neuron negative for Fos immunofluorescence. Graph shows the increase in the % of mDA neurons expressing Fos, following 10 μM sulpiride treatment. (mean ± SEM. N=5 cultures, P=0.0420, t=2.417 df=8; unpaired Student's t test). Scale bar is 20 μm. (**d**) Graph shows TH immunolabeling fluorescence intensity mean, together with distribution of individual neurons, following CTR or 10 μM Sulpiride treatments (mean ± SEM; N=3 cultures, P=0.1890, t=1.319 df=165; two-tailed unpaired Student's t test). *=P<0.05.

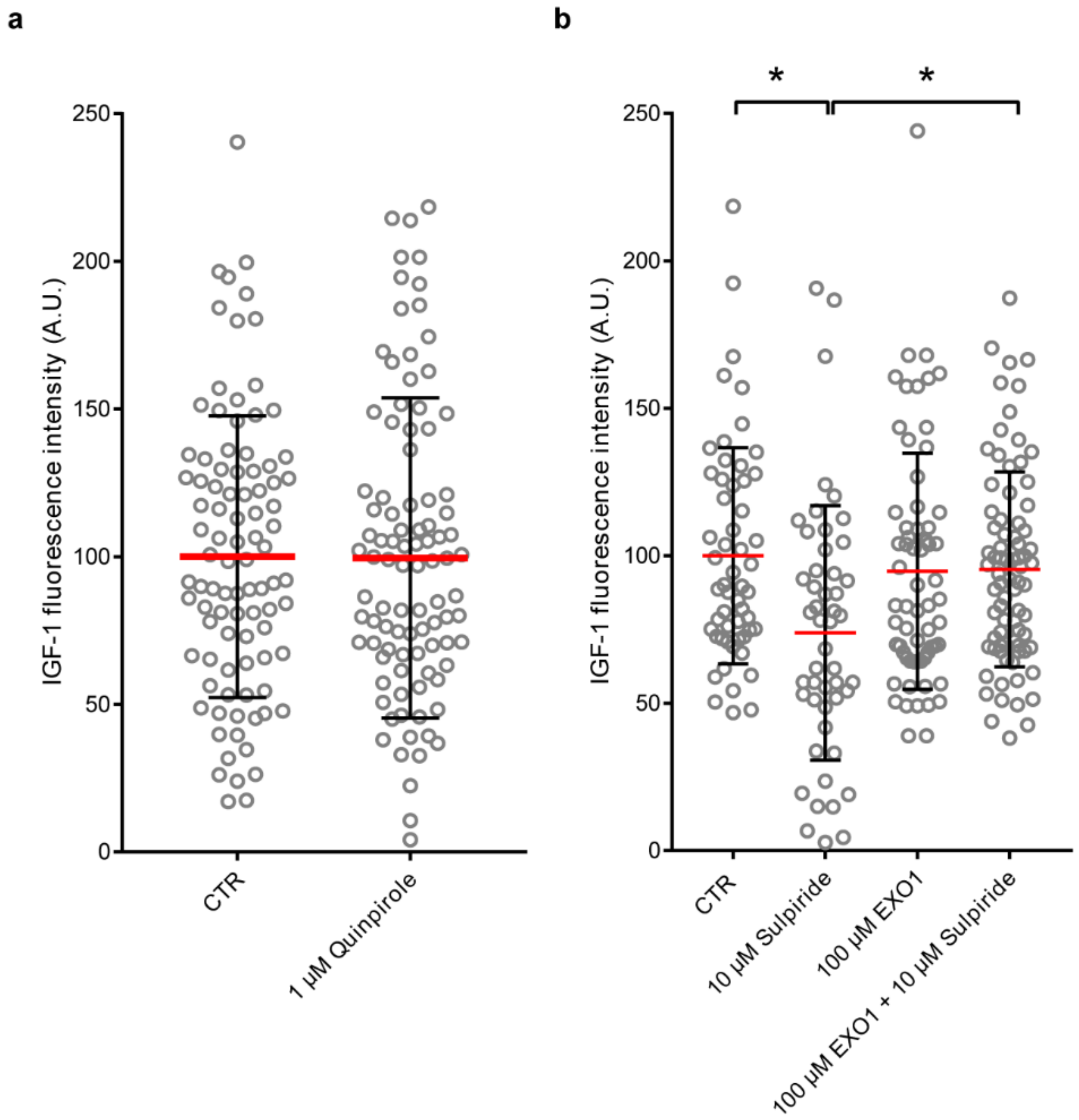

Fig. S3 Quinpirole does not modulate IGF-1 release and EXO1 blocks sulpiride-mediated IGF-1 release.

IGF-1 intracellular fluorescence intensity at the level of the cell bodies was quantified following different treatments, as in Fig. 2. (**a**) Graph shows mean ± SD, together with distribution of individual neuron, following 1 μM Quinpirole treatment (N=3, P=0.9533, t=0.05865 df=190; two-tailed unpaired Student's t test). (**b**) Graph shows mean ± SD, together with distribution of individual neuron, following 10 μM sulpiride, 100

μM EXO1 or 10 μM sulpiride + 100 μM EXO1 treatment (N=3, P CTR *vs*. 10 μM sulpiride = 0.0029, P 10 μM sulpiride *vs*. 10 μM sulpiride + 100 μM EXO1 = 0.0106, P CTR *vs*. 100 μM EXO1 = 0.8741; $F_{(3, 243)}$ = 4.893; one-way ANOVA with Tukey's correction for multiple comparisons). *=P<0.05.

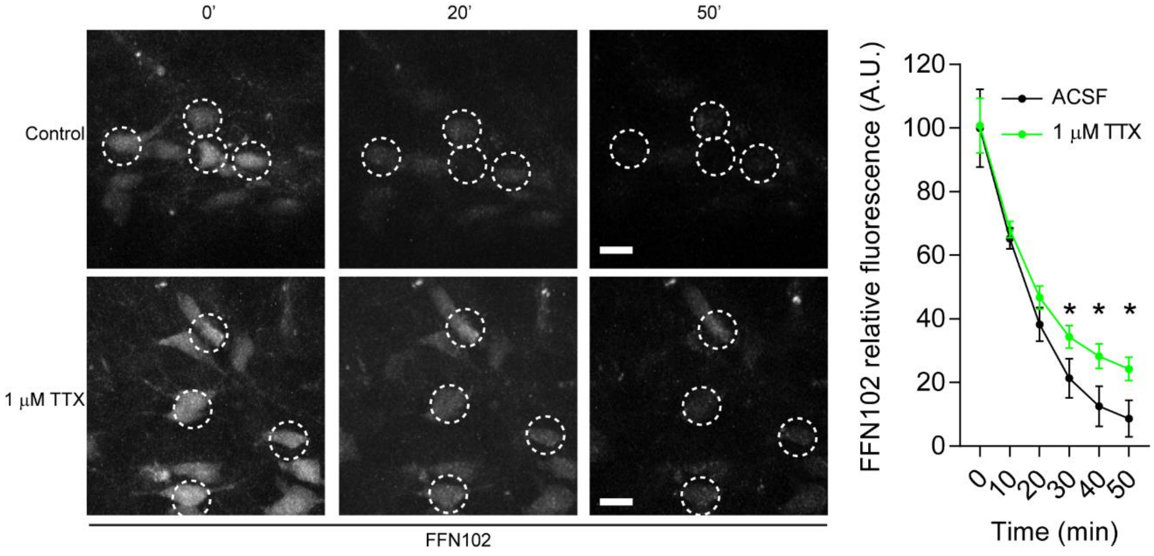

Fig. S4 TTX decreases FFN102 de-staining rate from the cell bodies of mDA neurons.

FFN102 release from the cell bodies of mDA neurons was quantified over time, with or without 1 μM TTX. Panels on the left show representative FFN102 fluorescence at different time points. Dotted ROIs show examples of mDA neurons, tracked over time. Graphs show normalised FFN102 fluorescence intensity over time (N=3, P ACSF *vs.* 1 μM TTX (T30 min, T40 min T50 min) = <0.012; $F_{(5, 720)}$ = 4.019; two way ANOVA with Sidak's correction for multiple comparisons). Time points show mean ± 95% CI. Scale bar is 20 μm.

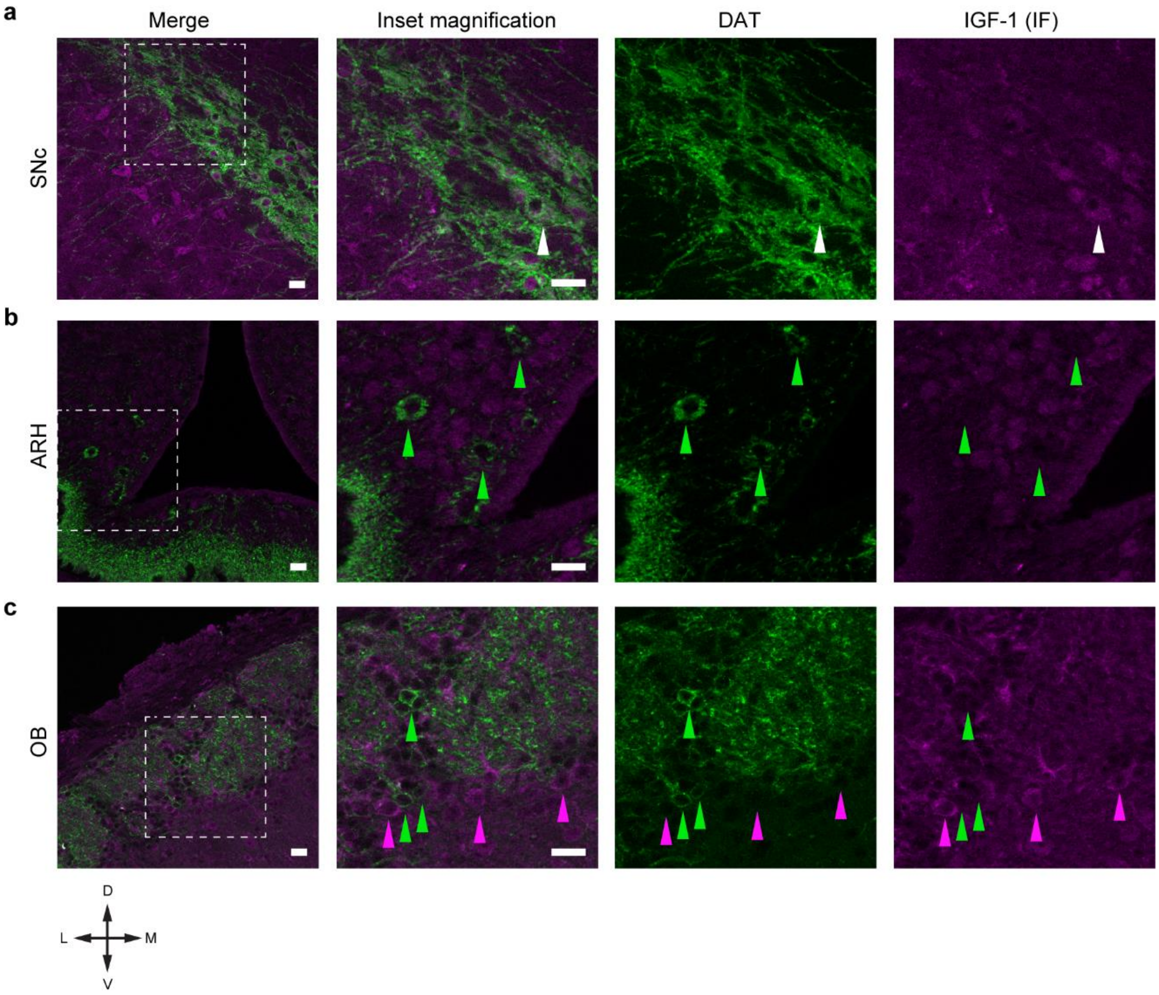

Fig. S5 Expression of IGF-1 in DAT expressing neurons.

This figure shows immunofluorescence for DAT and IGF-1 in different parts of the adult brain. (**a**) IGF-1 is expressed in DAT expressing neurons in the SNc. (**b**) DAT expressing neurons in the hypothalamus/arcuate nucleus (ARH) show no IGF-1 immunoreactivity. (**c**) IGF-1 is expressed in the olfactory bulb (OB), however it does not co-localise with DAT. White arrowheads point at co-localisation between DAT and IGF-1, green and magenta arrowheads point at DAT- and IGF-1-only expressing neurons, respectively. Scale bars are 20 μm.

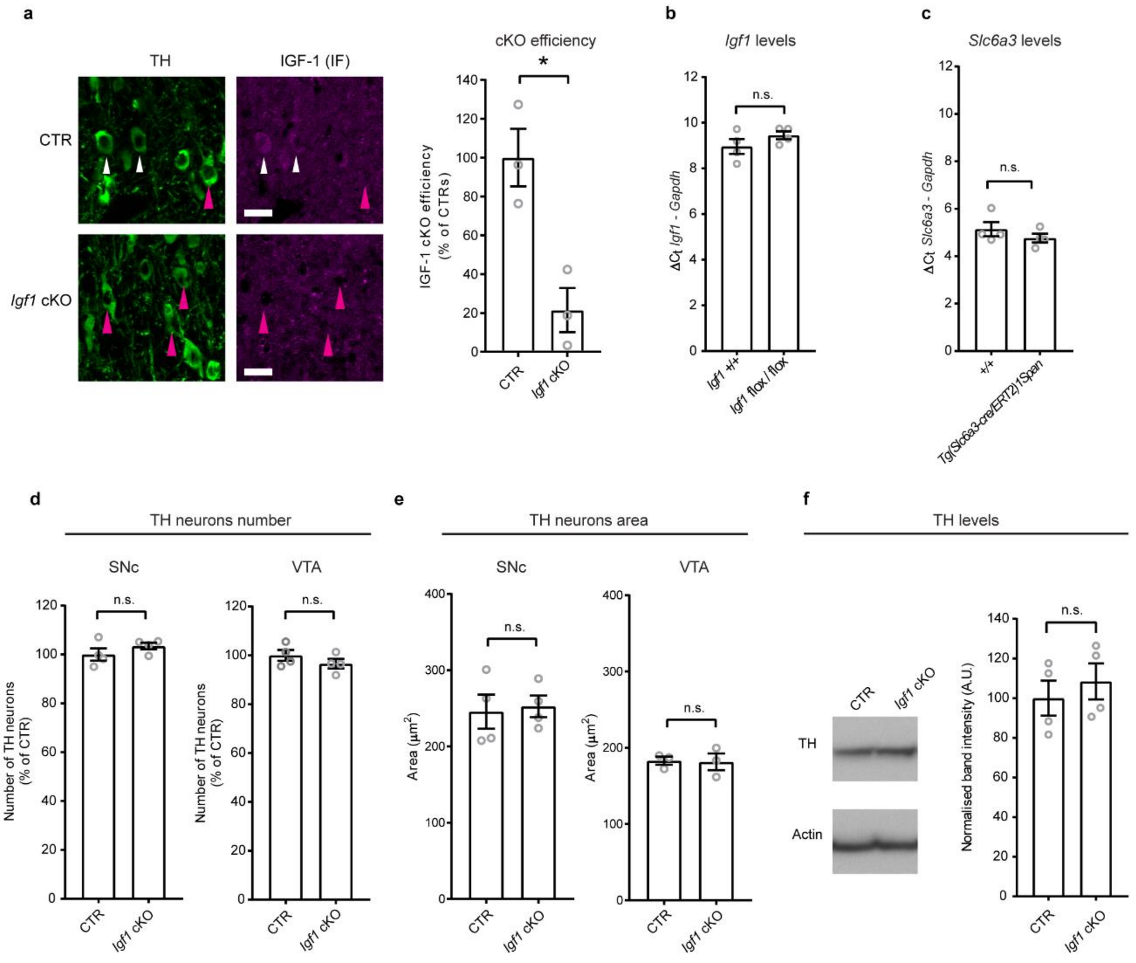

Fig. S6 *Igf1* cKO mice characterisation.

(**a**) Confirmation of tamoxifen induced recombination and IGF-1 deletion. Images shows representative immunofluorescence of TH neurons, expressing (white arrowheads) or not (magenta arrowheads) IGF-1, in CTR and *Igf1* cKO mice. Efficiency of IGF-1 deletion (expressed as % change *vs*. CTR) is quantified in the graphs (N=3; P=0.0137, t=4.204 df=4; two-tailed unpaired Student's t test). Scale bars are 20 μm. (**b**) *Igf1* floxed alleles are not hypomorphic. We quantified by RT-qPCR the amount of *Igf1* transcripts in ventral midbrain cDNA samples from wild-type and *Igf1* floxed mice, and did not detect changes in *Igf1* transcript levels (N=4, P=0.2307, t=1.334 df=6; two-tailed unpaired Student's t test). (**c**) The presence of transgene *Slc6a3*^{CreERT2} does not influence

endogenous *Slc6a3* gene expression. We quantified by RT-qPCR the amount of *Slc6a3* transcripts in ventral midbrain cDNA samples from wild-type and *Slc6a3*$^{CreERT2/+}$ expressing mice (N=4, P=0.3299, t=1.06 df=6; two-tailed unpaired Student's t test). (**d**) Number of TH expressing neurons (expressed as % change *vs*. CTR) in the SNc and VTA is not affected in *Igf1* cKO mice, compared to controls (SNc: N=4, P=0.2793, t=1.189 df=6; VTA: N=4, P=0.2892, t=1.162 df=6; two-tailed unpaired Student's t test). (**e**) Surface area, expressed as μm^2, of SNc and VTA neurons is not affected in *Igf1* cKO mice, compared to controls (SNc: N=4, P=0.8015, t=0.2628 df=6; VTA: N=3, P=0.9062, t=0.1254 df=4; two-tailed unpaired Student's t test). (**f**) Total TH protein content is not changed in *Igf1* cKO mice. Images show representative bands for TH and Actin, from ventral midbrain preparations, in CTR and *Igf1* cKO mice. Total TH levels were quantified by densitometry and normalised against Actin, used as loading control. Graphs shows quantification of bands density (N=4; P=0.5318, t=0.6633 df=6; two-tailed unpaired Student's t test). Bar graphs show mean ± SEM. *=P<0.05.

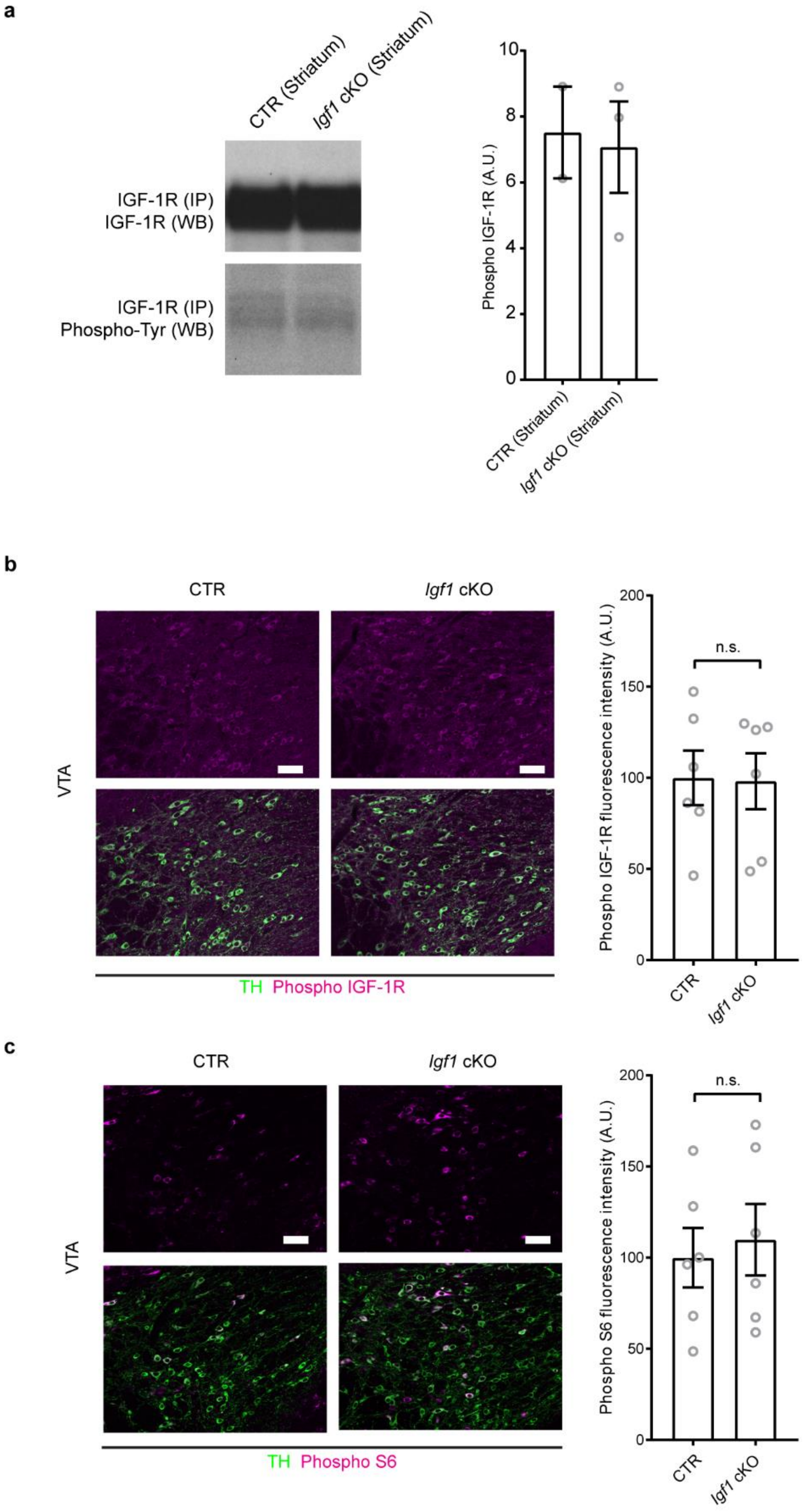

Fig. S7 IGF-1 signalling in *Igf1* cKO mice is not affected in the Striatum and VTA.

(**a**) Total IGF-1R was immunoprecipitated (IP) from the striatum of CTR and *Igf1* cKO mice and probed with anti phospho-Tyrosine antibody, to quantify its activation level. Panel on the left show representative bands of IP IGF-1R (IGF-1R IP, IGF-1R WB) and phospho-IGF-1R (IGF-1R IP, phospho-Tyr WB). Phospho-Tyrosine levels were normalised against total IGF-1R (CTR N=2, *Igf1* cKO N=3; P=0.8440, t=0.2144 df=3; two-tailed unpaired Student's t test). Upstream (IGF-1R) and downstream (S6 ribosomal protein) effectors of IGF-1 signalling were analysed. (**b**) Images show representative immunofluorescence for TH and phospho-IGF-1R in the VTA of control and *Igf1* cKO mice. Top panel shows phospho-IGF-1R only for clarity. Phospho-IGF-1R fluorescence intensity was not changed in TH positive VTA neurons in *Igf1* cKO mice compared to controls (N=6, P=0.9332, t=0.0859 df=10; two-tailed unpaired Student's t test). (**c**) Images show representative immunofluorescence for TH and phospho-S6 in the VTA of control and *Igf1* cKO mice. Top panel shows phospho S6 only for clarity. Phospho-S6 fluorescence intensity was not changed in the VTA of *Igf1* cKO mice, compared to controls (N=6, P=0.7074, t=0.3862 df=6; two-tailed unpaired Student's t test). Bar graphs show mean ± SEM. *=P<0.05.

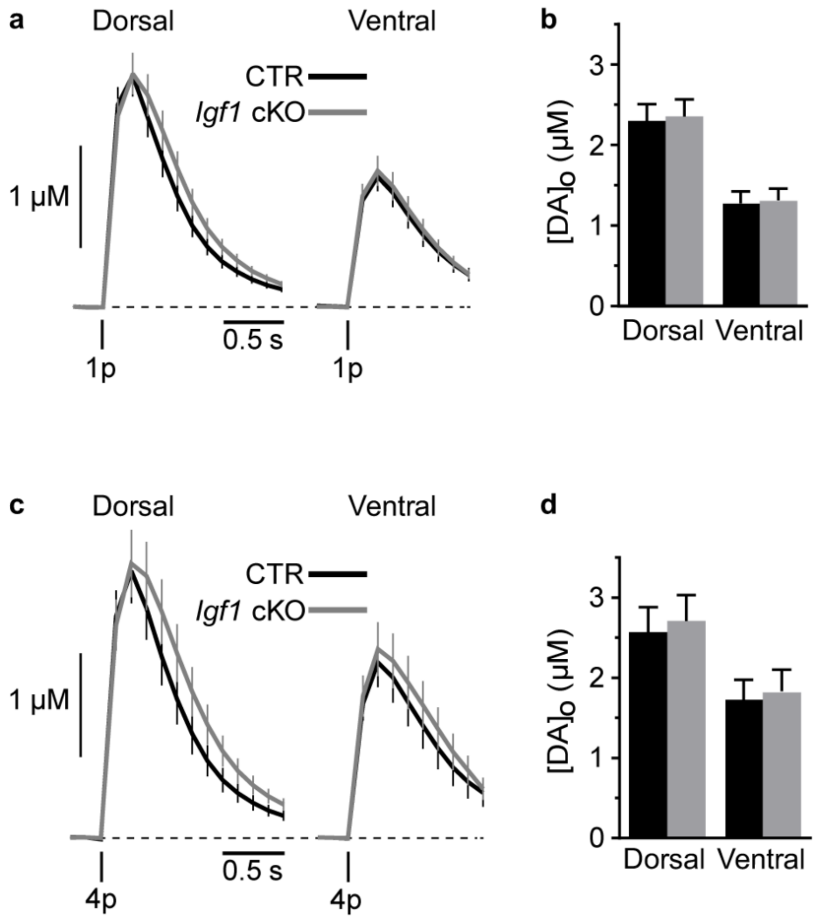

Fig. S8 Evoked striatal dopamine release per stimulus is unchanged in *Igf1* cKO mice.

(**a**, **c**) Mean release profiles (± SEM) of [DA]o in CTR and *Igf1* cKO animals following either 1p (**a**) or 4p 100 Hz (**c**) in dorsal and ventral striatal sites. (**b**, **d**) Mean peak evoked (± SEM) of [DA]o in CTR and *Igf1* cKO animals following either 1p (**b** – Dorsal striatum CTR *vs*. *Igf1* cKO: N=6, P=0.6658, t=0.4475, df=71; Ventral striatum CTR *vs*. Igf1 cKO: N=6, P=0.8057, t=0.2469, df=71; two-tailed paired Student's t test) or 4p 100 Hz (**d** - Dorsal striatum CTR *vs*. *Igf1* cKO: N=6, P=0.4273, t=0.8031, df=35; Ventral striatum CTR *vs*. *Igf1* cKO: N=6, P=6715, t=0.4276, df=35; two-tailed paired Student's t test).

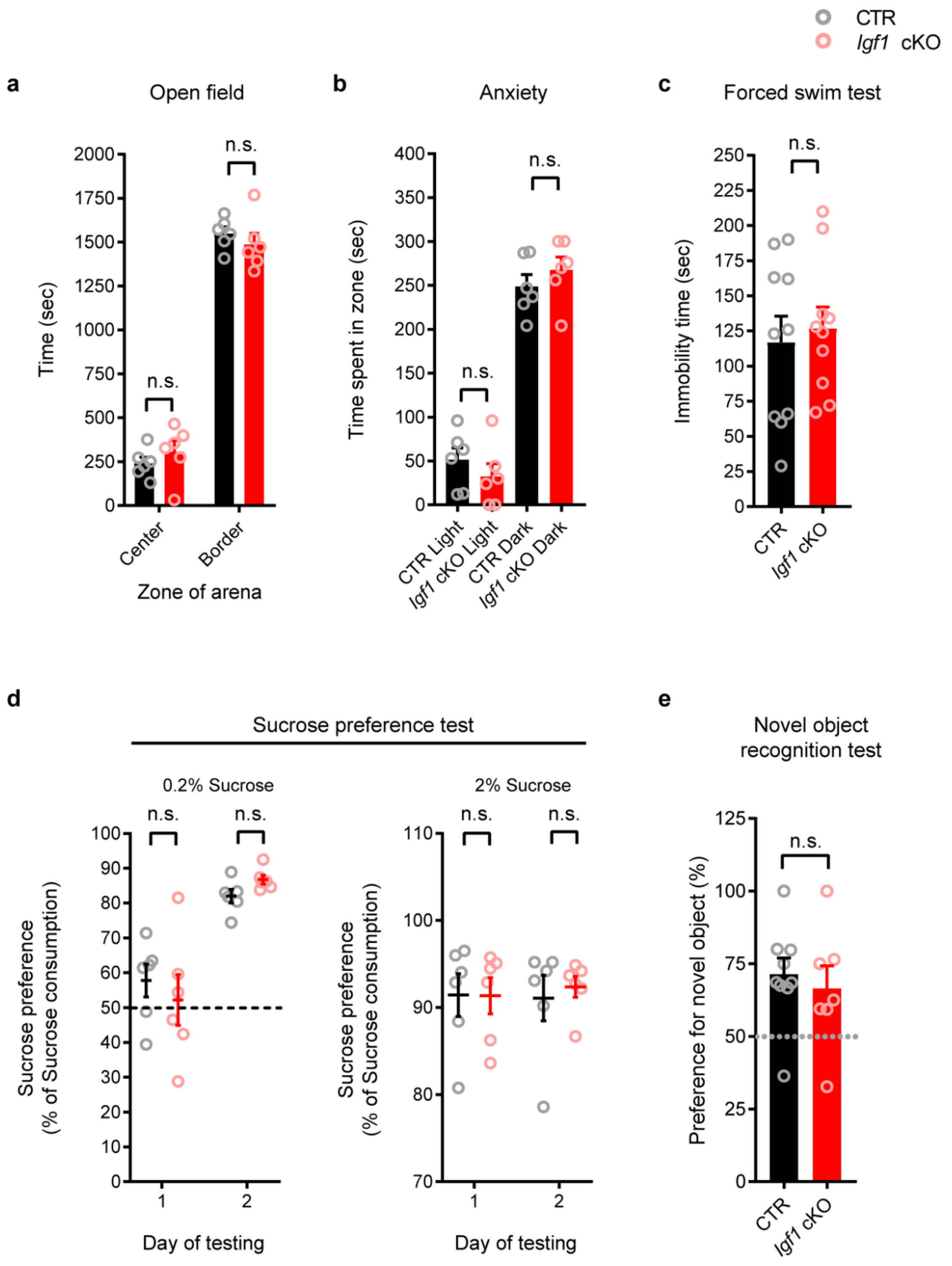

Fig. S9 *Igf1* cKO mice do not show differences in anxiety-, depression-like, anhedonia and novel object recognition behaviours, compared to control mice.

(**a**) Graphs show time spent (sec) in the centre (defined as a 28x28 cm square inside the arena) and border of the arena, over the 30 min trial. No differences were detected between controls and *Igf1* cKO mice (N=6; 5 males and 1 female per genotype. P CTR *vs. Igf1* cKO: Centre = 0.359, t=0.9609, df=10; Border = 0.4080, t=0.8636, df=10; two-tailed unpaired Student's t test). (**b**) Graphs show time spent (sec) in the light and dark sides of the arena. No differences were detected between controls and *Igf1* cKO mice (N=6; 5 males and 1 female per genotype. Light, Dark P=0.3624, t=0.9543 df=10; two-tailed unpaired Student's t test). (**c**) Graph shows time spent not moving (sec), during the forced swim test. No differences were detected between controls and *Igf1* cKO mice (N=10; 9 males and 1 female per genotype. P=0.6835, t=0.4143 df=18; two-tailed unpaired Student's t test) (**d**) Graphs show sucrose preference (% of sucrose solution consumption) over two days test; on different tests, mice were given the choice of water and 0.2% sucrose, or water and 2% sucrose. No differences were detected between controls and *Igf1* cKO mice (N=6; 5 males and 1 female per genotype. P CTR *vs. Igf1* cKO, 0.2% Sucrose test, Day1=0.7824 Day2=0.9234; $F_{(1, 10)}$ = 1.349; P CTR *vs. Igf1* cKO, 2% Sucrose test, Day1=>0.9999 Day2=>0.9999; $F_{(1, 10)}$ = 0.4617. Two-way ANOVA with Bonferroni's correction for multiple comparisons). (**e**) Graph shows preference for the novel object (calculated as percentage of total exploration time spent exploring the novel object). No differences were detected between controls and *Igf1* cKO mice (CTR N=9; 3 males and 6 females; *Igf1* cKO N=7; 3 males and 4 females. P=0.6098, t=0.5221 df=14; two-tailed unpaired Student's t test). All graphs show mean ± SEM.

References

1. Lin W, *et al.* (2009) Foxa1 and Foxa2 function both upstream of and cooperatively with Lmx1a and Lmx1b in a feedforward loop promoting mesodiencephalic dopaminergic neuron development. *Dev Biol* 333(2):386-396.
2. Rodriguez PC, *et al.* (2013) Fluorescent dopamine tracer resolves individual dopaminergic synapses and their activity in the brain. *Proc Natl Acad Sci U S A* 110(3):870-875.
3. Pristera A, *et al.* (2015) Transcription factors FOXA1 and FOXA2 maintain dopaminergic neuronal properties and control feeding behavior in adult mice. *Proc Natl Acad Sci U S A* 112(35):E4929-4938.
4. Franklin KBJ & Paxinos G (2012) *Paxinos and Franklin's the Mouse Brain in Stereotaxic Coordinates* p 360.
